# Top-emitting thermally activated delayed fluorescence organic light-emitting devices with weak light-matter coupling

**DOI:** 10.1038/s41377-021-00559-w

**Published:** 2021-06-03

**Authors:** Chunxiu Zang, Shihao Liu, Mengxin Xu, Ruifang Wang, Chen Cao, Zelin Zhu, Jiaming Zhang, Hui Wang, Letian Zhang, Wenfa Xie, Chun-Sing Lee

**Affiliations:** 1 State key Laboratory of Integrated Optoelectronics, College of Electronics Science and Engineering, Jilin University, 130012 Changchun, China; 2 Center of Super-Diamond and Advanced Films (COSDAF) and Department of Chemistry, City University of Hong Kong, 999077 Hong Kong SAR, China

**Keywords:** Organic LEDs, Photonic devices

## Abstract

Resonance interaction between a molecular transition and a confined electromagnetic field can lead to weak or strong light-matter coupling. Considering the substantial exciton–phonon coupling in thermally activated delayed fluorescence (TADF) materials, it is thus interesting to explore whether weak light-matter coupling can be used to redistribute optical density of states and to change the rate of radiative decay. Here, we demonstrate that the emission distribution of TADF emitters can be reshaped and narrowed in a top-emitting organic light-emitting device (OLED) with a weakly coupled microcavity. The Purcell effect of weak microcavity is found to be different for TADF emitters with different molecular orientations. We demonstrate that radiative rates of the TADF emitters with vertical orientation can be substantial increased in weakly coupled organic microcavity. These observations can enhance external quantum efficiencies, reduce efficiency roll-off, and improve color-purities of TADF OLEDs, especially for emitters without highly horizontal orientation.

## Abstract (web summary)

This work not only presents photophysical processes of thermally activated delayed fluorescent emitters in a weak Fabry-Pérot cavity, but also establishes a connection between emitter orientation and Purcell effect.

## Graphical abstract

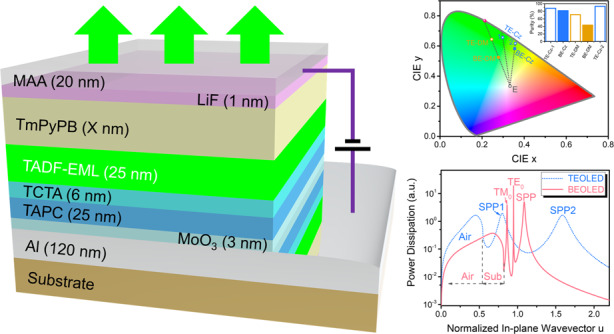

## Introduction

Thermally activated delayed fluorescence (TADF) materials have been widely considered as the third-generation emitters for organic light-emitting devices (OLEDs). Although they only utilize singlet excitons for radiation, TADF emitters can flip triplet (T) excitons to singlet (S) excitons via reverse intersystem crossing (RISC)^1–9^. However, due to the separated molecular orbitals, emissions of TADF emitters originate from intramolecular charge transfer, and easily undergo geometric relaxation, resulting in considerable exciton–phonon coupling^10–19^. Therefore, TADF emitters typically display broad emission spectra. While this broad emission is a great advantage for lighting applications, it is considered to have inadequate color purity for display applications.

To address the color purity issue, Hatakeyama et al.^20^ proposed a novel design of TADF molecules through multiple resonance effect^21^. Using the strategy, multiple TADF emitters with narrowband emission spectra have been developed^22–25^. In additional to novel material design, another approach to overcome the color purity issue might involve the light-matter coupling to redistribute the density of optical modes. A Fabry–Pérot (FP) cavity consisting of two opposing reflective mirrors is a versatile method for introducing light-matter coupling and then redistribute optical energy at selected wavelengths through building up a specific resonance condition^26–28^. In the FP cavities with different quality factors (Q factors), interactions between photonic and electronic excitations can be classified as weak or strong couplings^29^. For TADF emitters, Stéphane and colleagues have studied their RISC rate in strongly coupled microcavities consisting of two highly reflective silver mirrors^26^. However, for display applications, the weak light-matter coupling in a cavity (*Q* factor < 10) consisting of one highly reflective mirror and one reflective/semitransparent mirror is more important because of the requirement for high light extraction^27^. Furthermore, influences of the multi-beam interference and the Purcell effect on TADF emitters in a weakly light-matter coupled cavity are still unclear and deserve to be investigated.

Here, we report a simple but effective strategy for designing high color-purity and efficient TADF OLEDs by using a weakly coupled organic microcavity (*Q* factor < 9.6). In this work, top-emitting TADF OLEDs were prepared with a highly reflective anode Al (120 nm) and a reflective/semitransparent bilayer cathode Mg: 10 wt% Ag (1 nm)/Ag (19 nm) (MAA, 20 nm). We find that such TADF OLEDs show narrowed emission spectra with nearly half full-width at half maximums (FWHMs) of the conventional devices under the effects of multi-beam and wide-angle interferences. It is also demonstrated that the radiative recombination rate of TADF emitters can be efficiently accelerated by the Purcell effect, and the intrinsic radiative efficiency of the TADF emitters can thus be substantially improved. It also shows that while the substrate and waveguide modes are absent, a higher outcoupling efficiency can still be achieved for these top-emitting TADF devices by using a TADF emitter with a higher horizontal dipole ratio. By combining the faster radiative recombination rate, higher outcoupling efficiency and improved radiative efficiency, the external quantum efficiencies (EQEs) of the top-emitting TADF OLEDs are enhanced by over 60% comparing with the conventional TADF devices.

## Results

### Broad emission of the conventional TADF OLEDs

**Fig. 1:**
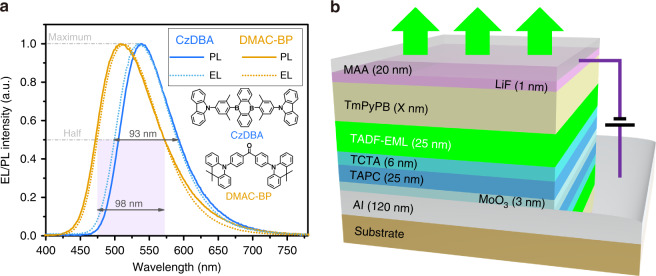
Broad emission of TADF emitters and a top-emitting structure with weak light-matter coupling. **a** Molecular structure and PL spectra (solid lines) of the CzDBA and DMAC-BP TADF emitters, and EL spectra (dotted lines) of their corresponding conventional bottom-emitting devices BE-Cz and BE-DM. **b** Device structure of the proposed top-emitting TADF devices.

Two green TADF emitters 9,10-bis(4-(9H-carbazol-9-yl)-2,6-dimethylphenyl)-9,10-diboraanthracene (CzDBA) and bis (4-(9, 9-dimethylacridin-10 (9H)-yl) phenyl) methanone (DMAC-BP) are used in this work. Photoluminescence (PL) spectra of CzDBA and DMAC-BP (in toluene, 1 mg/mil) are shown in Fig. 1a (solid lines). CzDBA has a PL quantum yield (PLQY) of ~100% and a horizontal dipole ratio Θ of 84% in its thin film^30^, while DMAC-BP shows a PLQY of ~86% and an isotropic dipole distribution^31^. Because of their different photophysical properties, the two TADF emitters are expected to experience different effects from the weak light-matter coupling. With these emitters, two conventional bottom-emitting TADF OLEDs with a device structure of ITO (150 nm)/MoO_3_ (3 nm)/TAPC (25 nm)/TCTA (6 nm)/CBP:10 wt% CzDBA or CBP:10 wt% DMAC-BP (25 nm)/TmPyPB (40 nm)/LiF (1 nm)/Mg:10 wt% Ag (120 nm) are fabricated and marked as devices BE-Cz and BE-DM, respectively. Full names of the used compounds are given in the experimental section. Electroluminescent (EL) spectra of devices BE-Cz and BE-DM are also shown in Fig. 1a (dotted lines). Devices BE-Cz and BE-DM show similar broad EL emissions to the corresponding PL emissions of their TADF emitters. As we see from Fig. 1a that all the FWHMs of their spectra are close to 100 nm.

### Top-emitting TADF OLEDs with narrow emission and high color-purity

The weak light-matter coupling, which is also called as weak microcavity effect, has been demonstrated to narrow the emission spectra of inorganic and organic emitters. Thus, top-emitting TADF OLEDs with a weakly coupled optical cavity are proposed as shown in Fig. 1b. A highly reflective anode Al (120 nm) and a reflective/semitransparent bilayer cathode Mg: 10 wt% Ag (1 nm)/Ag (19 nm) (MAA, 20 nm) are used to form the cavity, and their reflectivity and transmittance characteristics are shown in Fig. S1. In the conventional devices BE-Cz and BE-DM, a transparent anode ITO with a transmittance of 95% (@540 nm from the incident medium of organic to the exit medium of glass, Fig. S1a) is used, and its reflectivity is only 5% (@540 nm, Fig. S1b). Microcavity effect is considered to be negligible in these conventional devices, as we see their spectral characteristics in the Figs. 1a and S2. On the other hand, the MAA cathode of the top-emitting TADF OLEDs has a considerable reflectivity (54%@540 nm, Fig. S1b) and a considerable transmittance (45%@540 nm, Fig. S1a). The reflective/semitransparent MAA cathode and the highly reflective Al anode (87%@540 nm, Fig. S1b) can thus form an optical cavity in which light will reflect at the organic/MAA interface leading to both multiple beam and wide-angle interferences.

To evaluate the MAA-Al cavity, an intrinsic cavity emission spectrum C(*λ*) is defined as the spectral emission intensity emitted from an optical cavity when the emitter in the cavity has a unity emission intensity [*I*_0_(*λ*) = 1] for all wavelengths. C(*λ*) can be calculated by using a classical theoretical approach (Supplementary Note 1)^32^. Figure S3 shows a calculated C(*λ*) of MAA-Al cavities with various cavity lengths. It can be seen from the cavity emission spectra that the resonant wavelength *λ*_r_ of the cavities ranges increases from 470 to 576 nm as the cavity length increases from 80 to 110 nm. From these data, it can be confirmed that the *Q* factors of all these cavities are below 9.6 ($Q = \frac{{\lambda _r}}{{{\Delta} \lambda }}$, Δ*λ* is the resonance width). Thus, all these MAA-Al cavities are weakly coupled cavities for the two green emitters CzDBA and DMAC-BP.

**Fig. 2:**
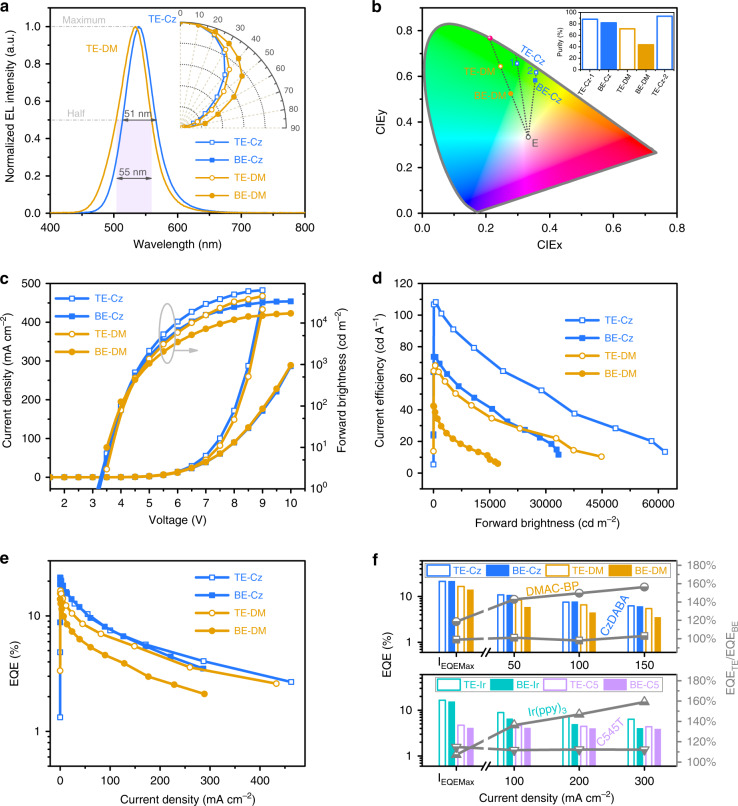
Device performances of top-emitting and bottom-emitting TADF OLEDs. **a** EL spectra and EL emission intensity angle distribution, **b** CIE coordinates and excitation purity, **c** current density–voltage-forward brightness characteristics, **d** current efficiency-forward brightness and **e** EQE-current density characteristics of devices BE-Cz, BE-DM, TE-Cz, and TE-DM. **f** The values of the EQE of the top-emitting and conventional devices with CzDBA, DMAC-BP, Ir(ppy)_3_, and C545T, and their EQE_TE_/EQE_BE_ ratios at different current densities (*I*_EQEMax_ corresponds to the current density of the maximum EQE).

Two green top-emitting TADF devices TE-Cz and TE-DM are then prepared using a weakly coupled cavity (Fig. 1b). Except the two electrodes, the top-emitting devices TE-Cz and TE-DM respectively have the same device structure as their corresponding conventional devices BE-Cz and BE-DM. As shown in Fig. 2a, b, devices TE-Cz and TE-DM show much narrower EL spectra and higher color purity compared with the conventional devices. FWHMs of the EL spectra of devices TE-Cz and TE-DM are respectively 51 nm and 55 nm, which are nearly half of those of the conventional devices. Top-emitting devices with the same structure but different cavity lengths are prepared by only changing the thickness (X) of the TmPyPB layer. Their EL emission spectra (solid lines in Fig. S4a, b) can be well fitted by using the product (dot lines in Fig. S4a, b) of the PL emission spectra of the TADF emitters and the cavity emission spectra of the MAA-Al cavities. This confirms that the spectra narrowing is indeed due to the multiple beam and wide-angle interferences.

Excitation purity of a color is defined by how close its coordinates to the edge of the CIE color space. For example, excitation purity of device TE-DM is determined by the ratio of (distance between color coordinates of TE-DM to a standard illuminant point, E) to (distance between the spectrum locus (solid pink sphere) and E point). While devices TE-DM and BE-DM have the same dominant wavelength (537 nm, refer to wavelength of the solid pink sphere in Fig. 2b), device TE-DM has a much higher color purity (71%) comparing to that of device BE-DM (43%). On the other hand, because of the intrinsic emission of CzDBA, device BE-Cz already has a high color purity of 82%. Nevertheless, device TE-Cz (spot 1 in Fig. 2b) still shows an improvement in the color purity (88%). However, it can be noted that device BE-Cz and device TE-Cz have different dominant wavelengths. To facilitate a more direct comparison, another TE-Cz (spot 2) device is prepared by changing X to 45.5 nm such that it has the same dominant wavelength as device BE-Cz. It is interesting to note that this further enhance the color purity of the top-emitting device (spot 2) to 93%.

### Device performances of the top-emitting TADF OLEDs

Weak light-matter coupling is also known for suppressing the undesired coupling of emitted photons to waveguided modes and enhance the efficiencies of thin-film devices. We thus investigate the current density–voltage-forward brightness (Fig. 2c), current efficiency-forward brightness (Fig. 2d), and EQE-current density (Fig. 2e) characteristics of the four devices. It can be noted that the current densities (Fig. 2c) of the top-emitting devices TE-Cz and TE-DM (empty symbols) are higher than those of the conventional bottom-emitting devices BE-Cz and BE-DM (solid symbols). This should not be attributed to the carrier injection issue because ITO/MoO_3_, Al/MoO_3_ and LiF/Mg:Ag have already been demonstrated as quasi-ohmic contacts in many previous reports^33–35^. We then simulate the voltage-current density characteristics by using a series resistance limited current model, and find that the current difference can thus be clearly clarified by the resistance difference between the ITO (27.7 Ω/□) and the MAA (2.6 Ω/□) (Fig. S5).

Figure 2d shows that the top-emitting devices TE-Cz and TE-DM show much higher current efficiencies (empty symbols) than their corresponding conventional devices BE-Cz and BE-DM (solid symbols). In fact, the current efficiency in candelas per amp (cd A^−1^) is used to quantify the properties of an OLED for display applications and measured by the ratio of the brightness (cd m^−2^) to the current density (mA cm^−2^)^36^. The improvements of the current efficiencies are partially due to the enhanced forward brightness (Fig. 2c) of the top-emitting devices, which is known as a result of the multiple beam and wide-angle interferences in the weakly coupled cavity (see the emission intensity angular distributions in Fig. 2a). On the other hand, device TE-Cz shows a similar EQE-current density characteristic (Fig. 2e) to that of device BE-Cz, while device TE-DM does show much higher EQEs (Fig. 2e) than device BE-DM. It indicates that the CzDBA and DMAC-BP emitters perform quite differently in the weakly coupled cavity. To clarify the reasons, top-emitting devices (TE-C5 and TE-Ir) and conventional bottom-emitting devices (BE-C5 and BE-Ir) with respectively a conventional fluorescent (C545T) and a conventional phosphorescent (Ir(ppy)_3_) emitters are prepared. C545T is 10-(2-Benzothiazolyl)-2,3,6,7-tetrahydro-1,1,7,7-tetramethyl-1H,5H,11H-^1^benzopyrano[6,7,8-ij]quinolizin-11-one and Ir(ppy)_3_ is tris(2-phenylpyridine)iridium(III). Characteristics of the devices with C545T and Ir(ppy)_3_ are given in the Fig. S6. We then summarized the EQEs of the above devices in the Fig. 2f and Table S1. The ratios of the EQEs (EQE_TE_) of the top-emitting devices to those (EQE_BE_) of their corresponding conventional devices at different current densities are also calculated (see the solid lines in Fig. 2f). As the current density increase, EQE_TE_/EQE_BE_ of the CzDBA-based and the C545T-based devices are not sensitive to the current density. On the other hand, EQE_TE_/EQE_BE_ of the DMAC-BP-based and the Ir(ppy)_3_-based devices increase with the current density. These differences are further analyzed by separately considering the effects of the microcavity on outcoupling and radiative efficiencies.

### Outcoupling efficiencies of the top-emitting TADF OLEDs

EQE of an OLED can be express with the equation:^37^

1 $${\mathrm{EQE}} = {\mathrm{IQE}} \cdot \eta _{{\mathrm{out}}} = \gamma \cdot \eta _{{\mathrm{rad}}} \cdot \eta _{{\mathrm{out}}}$$

where, IQE is the internal quantum efficiency. *γ* is the ratio of generated excitons (singlets for traditional fluorescence emitters) to the number of injected electron-hole pairs. *η*_rad_ is the intrinsic radiative efficiency of the emitter, and *η*_out_ is the outcoupling efficiency. As discussed above, the different current densities of the top-emitting and the bottom-emitting devices are mainly due to the resistance difference between ITO and MAA electrodes (Fig. S5). This suggests that the current balance (ie. electron vs hole currents) and thus *γ* in the two types of devices are not affected.

We then compare the outcoupling efficiencies *η*_out_ of the top-emitting devices and the conventional devices. The power dissipation spectrum is a powerful tool for analyzing different energy dissipation routes of an emitter in a multilayer system^38,39^. In fact, the power dissipation spectrum is mainly determined by the multilayer structure and the refractive indexes of the layers (Supplementary Note 2). For the four types of devices studied here, the top-emitting OLEDs (TEOLEDs) and the conventional bottom-emitting OLEDs (BEOLEDs) have electrodes of different reflectivity. Their organic multilayers are the same except the emitting layers. Figure S7 shows that the four emitting layers actually have very similar refractive indexes. With the small differences in the organic layers, the electrodes and the substrate are expected to have the dominating effects on the power dissipation spectrum. Nevertheless, Alq_3_ is used as the host of the C545T-doped emitting layer, while the CBP is used as the host of the other three types of emitting layers. Because of different transport properties, the C545T-doped emitting layers have different exciton distribution profiles comparing with the other three types of emitting layers. We here take the refractive index and the exciton distribution profiles (with refer to the profiles in the literature^40^) of the CBP: 10 wt% CzDBA as representatives for the subsequent calculations for the TEOLED and the BEOLED respectively. As regards C545T-based devices, their calculations are conducted separately.

**Fig. 3:**
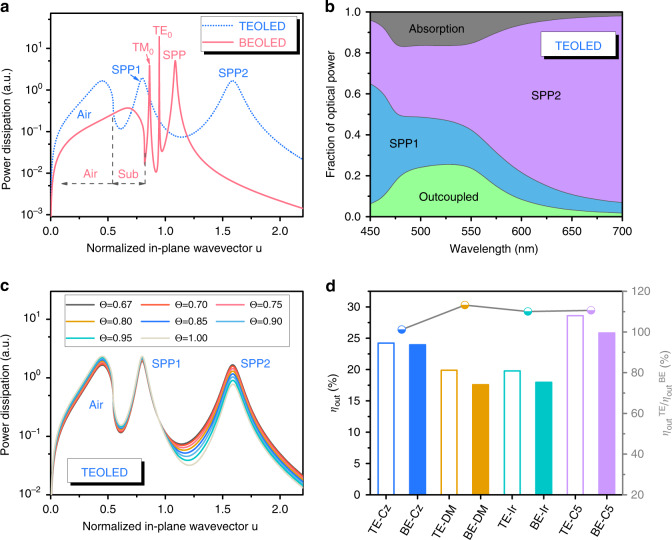
Power dissipation spectra and outcoupling efficiencies of OLEDs with weak microcavities. **a** Power dissipation spectra at *λ* = 540 nm for the conventional devices and the top-emitting devices, the in-plane wavevector u is normalized with respect to propagation in the emitting layer. **b** Distribution of all optical loss channels in the top-emitting devices. **c** Power dissipation spectra of organic emitters with different horizontal dipole ratio Θ in the top-emitting devices. **d** Outcoupling efficiency *η*_out_ of the top-emitting devices and the conventional devices, and their ratios *η*_out_^TE^/*η*_out_^BE^.

Figure 3a shows the power dissipation spectra calculated for the TEOLED and the BEOLED by assuming that the emitters have isotropic emission properties (i.e. Θ = 0.67) at 540 nm. The peaks in the spectra correspond to the modes of the optical cavity coupled with the radiating emitters. According to Figs. 3a and S8, the radiation in the BEOLED is coupled with two different guided modes (a TM-polarized mode, TM_0_; and a TE-polarized mode, TE_0_) and a surface plasmon polariton (SPP) mode. Because only the dielectric constant of the Mg:Ag cathode has a large negative real part, the SPP mode in the BEOLED should be contributed only by the organic/Mg:Ag interface. Indeed the peak position of the SPP mode (*u* ~ 1.08) in Fig. S8 matches well to the peak position (*u* ~ 1.08) of the Org*/Mg:Ag multilayer in Fig. S9. Besides, due to total internal reflection at the glass/air interface, the radiation with *n*_air_/*n*_e_ < *u* < *n*_glass_/*n*_e_ (i.e. 0.546 < *u* < 0.787) will be confined into the glass substrate (substrate mode, Sub) and only the radiation with *u* < 0.546 can escape to the air (air mode, Air). On the other hand, as shown in Fig. 3a, radiation in the TEOLED shows different coupled modes. Based on the discussion in Supplementary Note 3 (Fig. S10), the peak at *u* ~ 0.80 (the blue line in Fig. 3a) is associated to the hybrid mode of the SPP at the organic/Al interface and the Fabry–Pérot (FP) cavity, while the peak at *u* ~ 1.58 is associated to the hybrid SPP mode of the organic/MAA/air multilayer structure. Thus, only air mode and two different SPP modes are observed in the TEOLED, while substrate mode and waveguided modes are absent.

Intensity of the peaks in the spectra also provides information on the coupling strength to each of the observed modes. Using the power dissipation spectra at different wavelengths (Fig. S11a, b), we can quantify the optical loss caused by the different modes and the outcoupling efficiency of the devices, as shown in Figs. 3b and S12. It can be seen that the TEOLED presents an outcoupling characteristic as a function of wavelength, while the BEOLED has similar outcoupling efficiencies from 450 to 700 nm. As seen in Figs. 3b and S11b, the wavelength-dependent outcoupling characteristic of the TEOLED should be related to the coupling to the SPP1 and the SPP2 modes.

In addition to wavelength of the photon, orientation of the emitter also has an important influence. We then repeat the calculation by replacing the isotropic emitter with emitters with different preferred orientations. It can be seen in Figs. 3c and S13 that the coupling to the SPP mode in the BEOLED and the SPP2 mode in TEOLED are obviously weakened by increasing the horizontal dipole ratio Θ of the emitter. It indicates that a high Θ is also beneficial to the outcoupling efficiency of the TEOLED. However, when the value of the Θ increases from 0.67 (isotropic) to 0.84, the outcoupling efficiency of the TEOLED only increases by 23.4% (Fig. S14), while that of the BEOLED increases by 41.6% (Fig. S14). This is ascribed to the high absorption losses (gray area in Fig. 3b and gray area in Fig. S12) of the MAA-Al cavity caused by the multiple beam interference and the absorption properties of the MAA and Al layers (Fig. S15). Besides, the influences of Θ on the outcoupling efficiency of the TEOLED are also wavelength-dependent (Fig. S16). It is also attributed to the effects of the weak light-matter coupling.

As noted above, by convoluting the power dissipation spectra with the PL spectra of the emitters, the overall outcoupling efficiencies of the devices shown in Fig. 2f are calculated (Fig. 3d). For the isotropic emitters (DMAC-BP and Ir(ppy)_3_)^31,41^, the outcoupling efficiencies of their top-emitting devices (TE-DM and TE-Ir) are nearly 10% higher than those of their conventional devices (BE-DM and BE-Ir). On the other hand, the films of CBP: 10 wt% CzDBA and Alq_3_: 1 wt% C545T have a preferred horizontal emission dipole with Θ = 0.84 and Θ = 0.86 respectively^30,42^. Thus, devices TE-Cz and BE-Cz show much higher outcoupling efficiencies (Fig. 3d) and EQEs (Fig. 2f) comparing with the devices with the isotropic emitters. It should also be noted that the outcoupling efficiency of device TE-Cz is similar to device BE-Cz. This actually accords with their similar EQE-current density characteristics (Fig. 2f). Besides, Alq_3_ is an electron transport material. Excitons are expected to accumulate at the interface between the emitting layer and TCTA in C545T-based devices. It will lead to a reduced SPP2 (SPP) loss (Fig. S17) and a higher outcoupling efficiency (Fig. 3d) of device TE-C5 (BE-C5), compared with the CBP-based devices. This analysis indicates that the adjustment of exciton distribution will further improve the outcoupling efficiency of TADF devices.

### Purcell effect in the top-emitting TADF OLEDs

**Fig. 4:**
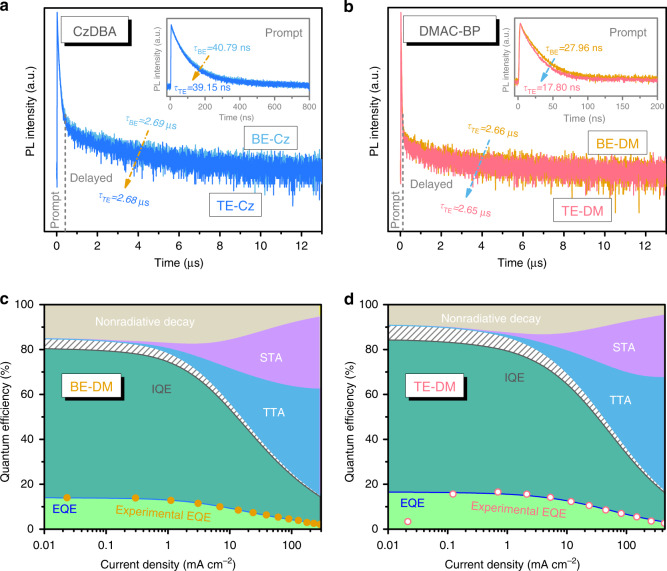
Purcell effect for TADF emitters with different horizontal orientations in weak microcavities. Transient PL decay characteristics of **a** the CzDBA in the devices TE-Cz and BE-Cz and **b** the DMAC-BP in the devices TE-DM and BE-DM. Theoretically calculated IQE, EQE, and EQE loss due to nonradiative decay, TTA and STA, **c** for device BE-DM and **d** for device TE-DM. Areas filled with stripes in **c** and **d** correspond to the EQE loss caused by the γ.

Apparently, the variation of the outcoupling efficiency only plays a part in the EQE improvement of device TE-DM comparing with device BE-DM. Therefore, the enhancement of the Purcell effect to the emission rate of the emitters should be considered. We then measured the transient PL decay characteristics of the organic emitters in the different devices. As shown in Fig. 4a, b, the peaks close to 0 μs correspond to the prompt component, followed by the slow delayed fluorescence due to RISC and re-emission. It can be seen that the delayed PL decay of the CzDBA and DMAC-BP emitters are similar in both the top-emitting and the bottom-emitting configurations. This result agrees with the conclusion proposed by Stéphane and colleagues that the RISC rate cannot be enhanced by strong light-matter coupling^26^.

On the other hand, we also observed that the emission rate of the TADF emitters can still be accelerated by the Purcell effect. As shown in the inset of Fig. 4b, the prompt PL decay of the DMAC-BP in device TE-DM (17.80 ns) becomes much faster than that of the DMAC-BP in device BE-DM (27.96 ns). The accelerated prompt PL decay of the DMAC-BP is considered as a result of the Purcell effect^43^. The faster PL decay can also be observed for the Ir(ppy)_3_ in device TE-Ir (Fig. S18). However, the prompt PL decays (see the inset of Fig. 4a) of the CzDBA are similar in device TE-Cz and device BE-Cz. It is also the same for the PL decays (Fig. S19) of the C545T in device TE-C5 and device BE-C5. It is considered to be due to the high horizontal emission dipole orientations of the CzDBA and the C545T. As proposed by Knight et al.^44^ the optical modes with polarizations normal to the planar microcavity cannot interact with the horizontal dipoles very well. Besides, our calculated results using the dipole model (Supplementary Note 2 and Fig. S20) also support the conclusions of Knight et al. This should be attributed to that an electrical dipole shows different radiation patterns in the vertical direction and the horizontal direction (see the inset of Fig. S20).

The intrinsic radiative efficiency *η*_rad_ (Eq. 1) of the emitters can be modified by the Purcell effect. The modified radiative efficiency *η*_rad′_ of the emitters is expressed as:^39^

2 $$\eta _{{\mathrm{rad}}^{\prime} } = \frac{{F_p\eta _{{\mathrm{rad}}}}}{{\left( {F_p - 1} \right)\eta _{{\mathrm{rad}}} + 1}}$$

As noted above, the spontaneous emission rate of the DMAC-BP is demonstrated to be accelerated by the Purcell effect. With the exciton lifetimes in Fig. 4b, a Purcell factor *F*_p_ of 1.66 is obtained, and the modified radiative efficiency *η*_rad′_ of the DMAC-BP in device TE-DM will thus be improved to 0.91. Besides, according to Eq. 2, the radiative efficiency of the CzDBA will be unchanged, because the PLQY of the CzDBA is a unity. Using the outcoupling efficiencies *η*_out_ (Fig. 3d), the radiative efficiencies *η*_rad_ (Table S2), and the maximum EQEs (Fig. 2f), we can obtain the values of the *γ* (Eq. 1), as shown in Fig. S21. It supports our previous conclusion that the *γ* is nearly same for the top-emitting devices and the conventional devices.

Furthermore, influences of the Purcell effect on the efficiency roll-off should also be considered to clarify the EQE improvement of device TE-DM (Fig. 2f). The significant EQE loss in typical TADF OLEDs have been attributed to singlet-triplet annihilation (STA) and triplet-triplet annihilation (TTA). We proceed to determine their relative contributions by analyzing densities of triplets (*n*_T_) and singlets (*n*_S_) at steady state which can be described as:^45^

3 $$0 = \frac{1}{4}k_{\mathrm{L}}n_{\mathrm{p}}^2 - \left( {k_{{\mathrm{ISC}}} + k_{\mathrm{s}} + k_{{\mathrm{ST}}}n_{\mathrm{T}}} \right)n_{\mathrm{s}} + k_{{\mathrm{RISC}}}n_{\mathrm{T}} + \frac{1}{8}k_{{\mathrm{TT}}}n_{\mathrm{T}}^2$$

4 $$0 = \frac{3}{4}k_{\mathrm{L}}n_{\mathrm{p}}^2 - \left( {k_{{\mathrm{RISC}}} + k_{\mathrm{T}}} \right)n_{\mathrm{T}} + k_{{\mathrm{ISC}}}n_{\mathrm{S}} - \frac{5}{8}k_{{\mathrm{TT}}}n_{\mathrm{T}}^2$$

5 $$n_{\mathrm{P}} = \sqrt {\frac{j}{{qwk_{\mathrm{L}}}}}$$

where, *j*, *q*, *w*, *k*_TT_, *k*_ST_, *k*_L_, *k*_T_ are respectively current density, elementary charge, width of the recombination zone, TTA rate, STA rate, Langevin recombination rate, and the reciprocal of triplet lifetime. Exciton radiation rate *k*_s_ will be replaced by a modified radiation rate k_s_′ in the top-emitting devices. Equations 3, 4, and 5 are solved by using the parameters (Table S3) with refer to the typical values in the literatures^45–47^. The solved values of *n*_T_ and *n*_S_ are shown in Fig. S22. The IQE of the devices can then be determined by IQE = *k*_S_*n*_S_/(*j*/*qw*), while EQE losses due to nonradiative decay, TTA and STA are respectively described as $k_{\mathrm{T}}n_{\mathrm{T}}$, $\frac{1}{2}k_{{\mathrm{TT}}}n_{\mathrm{T}}^2$ and $k_{{\mathrm{ST}}}n_{\mathrm{T}}n_{\mathrm{S}}$. The calculated IQE, EQE, and EQE losses due to nonradiative decay, TTA and STA of devices BE-DM and TE-DM are respectively shown in Fig. 4c, d. It can be seen that the EQE losses due to nonradiative decay, TTA and STA of device TE-DM become much smaller than those in device BE-DM under the same current densities. We should note that the radiation rates *k*_s_′ and *k*_s_ are the only variables (see Table S3) of the calculations for the EQE losses (the *k*_ISC_ is determined by the product of the PLQY and *k*_s_′ or *k*_s_). Thus, the reduced EQE losses (Fig. 4d) should be attributed to the accelerated spontaneous emission rate in the top-emitting device with the weak light-matter coupling. Similarly, because of the accelerated spontaneous emission rate, the EQE losses due to TTA and polaron-triplet annihilation (TPA) in device TE-Ir (Fig. S23a) are also reduced comparing with device BE-Ir (Fig. S23b). As a result, the combination of the enhanced outcoupling efficiency and the accelerated spontaneous emission rate can exactly clarify the EQE improvement of device TE-DM (Fig. 2f) comparing with device BE-DM.

Finally, there are typical angular color shifts in the top-emitting OLEDs with the weak light-matter coupling (Figs. S24 and S25). But it can be addressed by engineering the angle dependence of the metal reflection phase. For example, Joo et al.^27^ recently have proposed a nanopatterned metal mirrors for getting angle-stable OLED displays beyond 10,000 pixels per inch.

## Discussion

We prepared green top-emitting TADF OLEDs by using a highly reflective anode Al (120 nm) and a reflective/semitransparent bilayer cathode MAA (20 nm). The formed MAA-Al microcavities are demonstrated to be weakly coupled cavities (*Q* factors < 9.6) for green emitters. TADF emission from the MAA-Al microcavities can be reshaped and narrowed by the effects of the multiple beam interference and the wide-angle interference. Without coupling with the waveguided and substrate modes, the outcoupling efficiency can be enhanced for TADF emission from the top-emitting devices comparing with the conventional devices. We also observe that the spontaneous emission rate of TADF emitters is accelerated by the Purcell effect. These effects of the weak light-matter coupling can effectively improve the intrinsic radiative efficiency of TADF emitters, leading to an enhanced EQE and reduced efficiency roll-off of TADF OLEDs.

## Materials and methods

### Materials

Tris(2-phenylpyridine)iridium(III) [Ir(ppy)_3_] were purchased from Xi’an Polymer Light Technology Corp. MoO_3_, di-[4-(N, N-di-p-tolyl-amino)-phenyl]cyclohexane (TAPC), 4,4′,4″-Tris(carbazol-9-yl) triphenylamine (TCTA), 4,4′-Bis (carbazol-9-yl) biphenyl (CBP), 5,10-bis (4-(9H-carbazol-9-yl)-2,6-dimethylphenyl)-5,10-dihydroboranthrene (CzDBA), bis [4-(9,9-dimethyl-9,10-dihydroacridine) phenyl] methanone (DMAC-BP), 10-(2-Benzothiazolyl)-2,3,6,7-tetrahydro-1,1,7,7-tetramethyl-1H,5H,11H-(1)benzopyropyrano(6,7-8-I,j)quinolizin-11-one (C545T), 1,3,5-Tri[(3-pyridyl)-phen-3-yl] benzene (TmPyPB) and LiF of OLED grade were purchased from Lumtech Corp. All of the materials were directly used without further purification.

### OLED fabrication

ITO substrates (25 Ω sq^−1^) were cleaned by ultrasonic cleaning, dried at 120 °C, and then treated with air plasma for 5 min. All the organic layers and cathodes were then deposited in turn on the substrates via thermal evaporation. Evaporating rates of the organic materials, LiF, MAA, Al, and Mg:Ag are 1–2, 0.05–0.1, 0.05–0.1, 2–3, and 2–3 Å s^−1^, respectively. The effective device area is 10 mm^2^ for all the devices, determined by the overlap between the cathode and the anode.

### Device characterization

EL performances of all OLEDs were measured in atmosphere at room temperature by using a goniophotometric measurement system (Otsuka Electronics, Japan) consisting of a Keithley 2400 source, a MCPD 9800 spectrometer, a light receiving fiber and an integration sphere. Transient PL characteristics were collected by a time-resolved fluorescence spectrometer system (HORIBA, IHR320, Japan). Refractive indexes and film thicknesses of organic emitters were measured with an ellipsometer (J.A.Woollam, M-2000UI, USA). Power dissipation spectra were obtained by a home-made simulation software OptiXLED based on a classical electromagnetic approach^38,39^.

## Supplementary information

Supplementary Information
