# Supplementary material for: Top-emitting thermally activated delayed fluorescence organic light-emitting devices with weak light-matter coupling
Source: Light Sci Appl. 2021 Jun 3;10:116. doi: 10.1038/s41377-021-00559-w (PMC8175730; doi:10.1038/s41377-021-00559-w)
Supplement: Supplementary file 1 — Supplementary Information [file 41377_2021_559_MOESM1_ESM.docx]

Supplementary Information for

Top-emitting thermally activated delayed fluorescence organic light-emitting devices with weak light-matter coupling

Chunxiu Zang^1†^, Shihao Liu^1,2†*^, Mengxin Xu^1^, Ruifang Wang^2^, Chen Cao^2^, Zelin Zhu^2^, Jiaming Zhang^1^, Hui Wang^1^, Letian Zhang^1^, Wenfa Xie^1*^, Chun-Sing Lee^2*^

^1^State key Laboratory of Integrated Optoelectronics, College of Electronics Science and Engineering, Jilin University, Changchun, 130012, China

^2^Center of Super-Diamond and Advanced Films (COSDAF) and Department of Chemistry, City University of Hong Kong, Hong Kong SAR, 999077, China

*e-mail: [liushihao@jlu.edu.cn](mailto:liushihao@jlu.edu.cn), [xiewf@jlu.edu.cn](mailto:xiewf@jlu.edu.cn), [apcslee@cityu.edu.hk](mailto:apcslee@cityu.edu.hk)

^†^These authors contributed equally to this work.


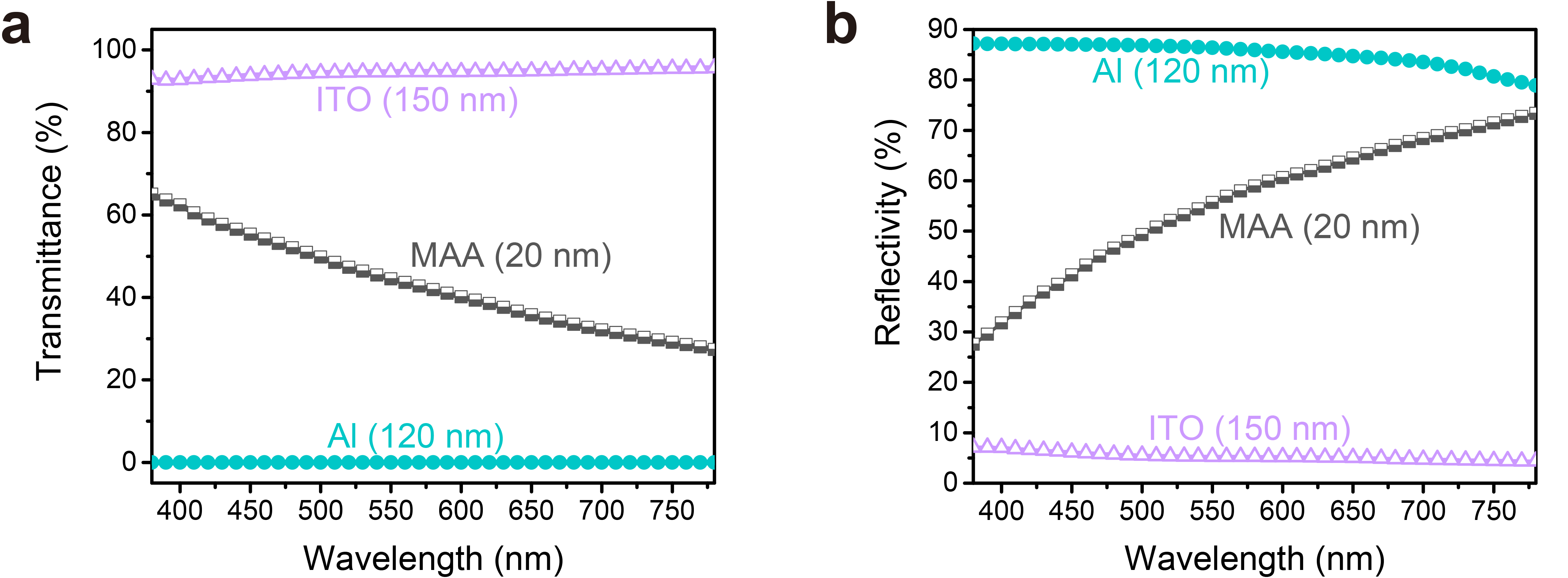


**Figure S1**. **(a)** Transmittance and **(b)** reflectivity characteristics of ITO transparent anode, Al reflective anode and MAA reflective/semitransparent cathode.


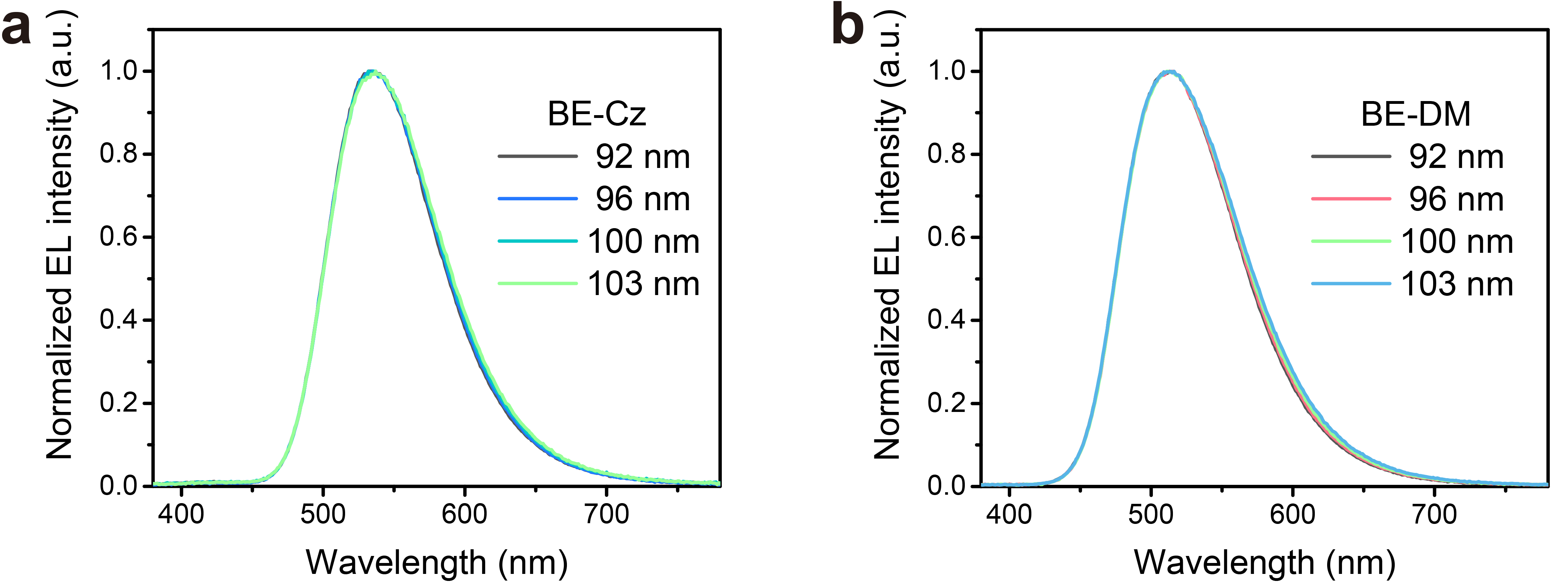


**Figure S2**. Normalized EL spectra of devices **(a)** BE-Cz and **(b)** BE-DM with different cavity lengths.


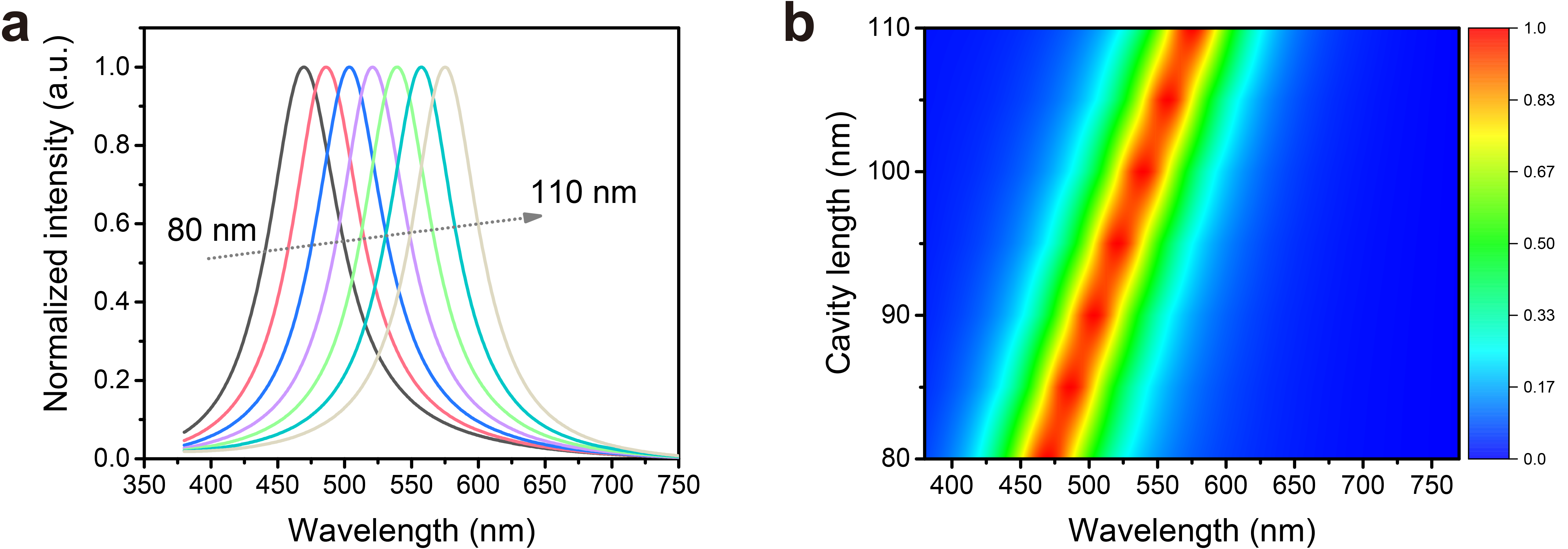


**Figure S3**. **(a)** Cavity emission spectra, C(λ), of MAA/Al optical cavities with various cavity lengths and **(b)** their 2D contour plots.

**Supplementary Note 1:**

The forward spectral emission intensity I(λ) from the optical cavity can be written as ^[S1, S2]^:

$I\left( \lambda\right)=\frac{T_{MAA}\left[ 1+R_{Al}+2\sqrt{R_{Al}}\cos\left( \frac{4\pi nz}{\lambda} \right) \right]}{1+R_{Al}R_{MAA}-2\sqrt{R_{Al}R_{MAA}}\cos\left( \frac{4\pi nL}{\lambda} \right)}I_{0}\left( \lambda\right)=C(\lambda)I_{0}\left( \lambda\right)$,

where, R_MAA_ (T_MAA_) and R_Al_ are the reflectivity (transmittance) of the MAA and Al electrodes, respectively. λ, n and z are respectively the wavelength, the refractive index and the distance from the emitting molecules to the highly refractive Al anode. I_0_(λ) is the spectral emission intensity of the radiating emitter in the free space. According to the above equation, the C(λ) is equal to the spectral emission intensity from the optical cavity when the radiating emitter has a unity emission intensity [I_0_(λ)=1]. As it is independent of the radiating emitter, the C(λ) is considered as an intrinsic cavity emission spectrum of the optical cavity.


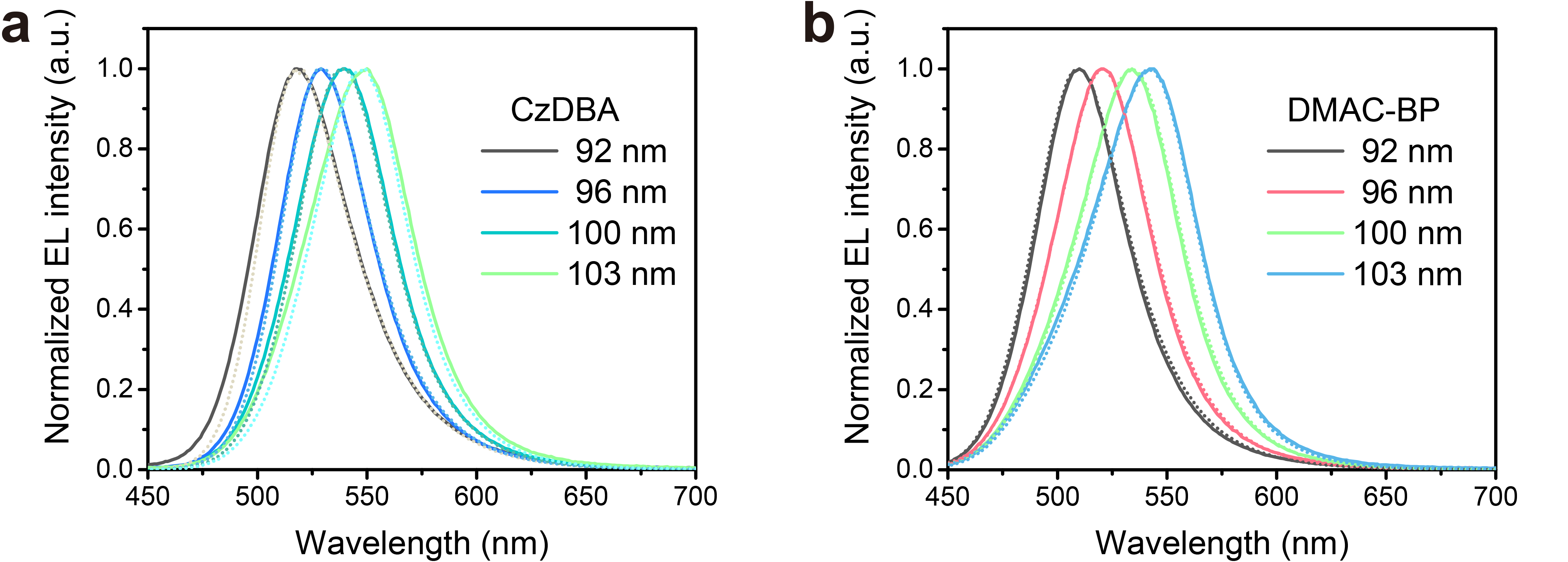


**Figure S4**. Normalized EL spectra of **(a)** the CzDBA-based and **(b)** the DMAC-BP-based top-emitting devices with different cavity lengths. The solid lines and dash lines are respectively the experimental and simulated results.


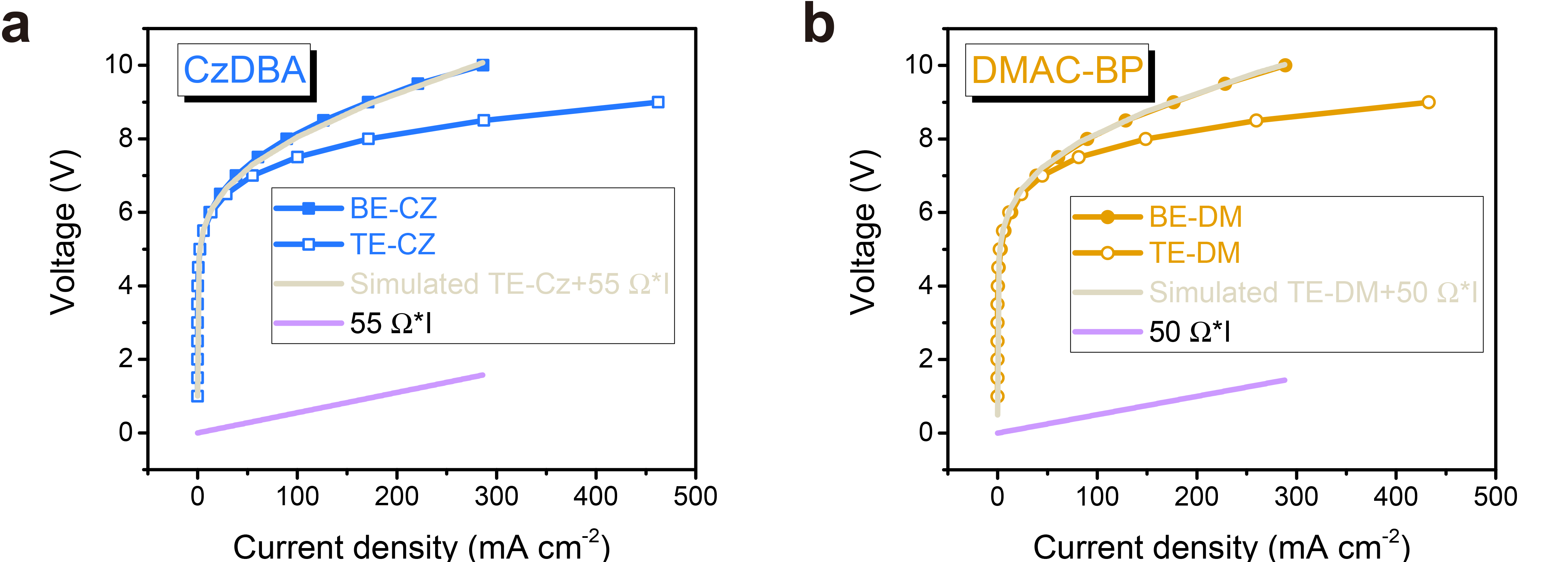


**Figure S5**. Voltage-current density characteristics of **(a)** CzDBA-based and **(b)** DMAC-based devices. Here, I is the current and is equal to the product of current density and injection area (~0.1 cm^2^).


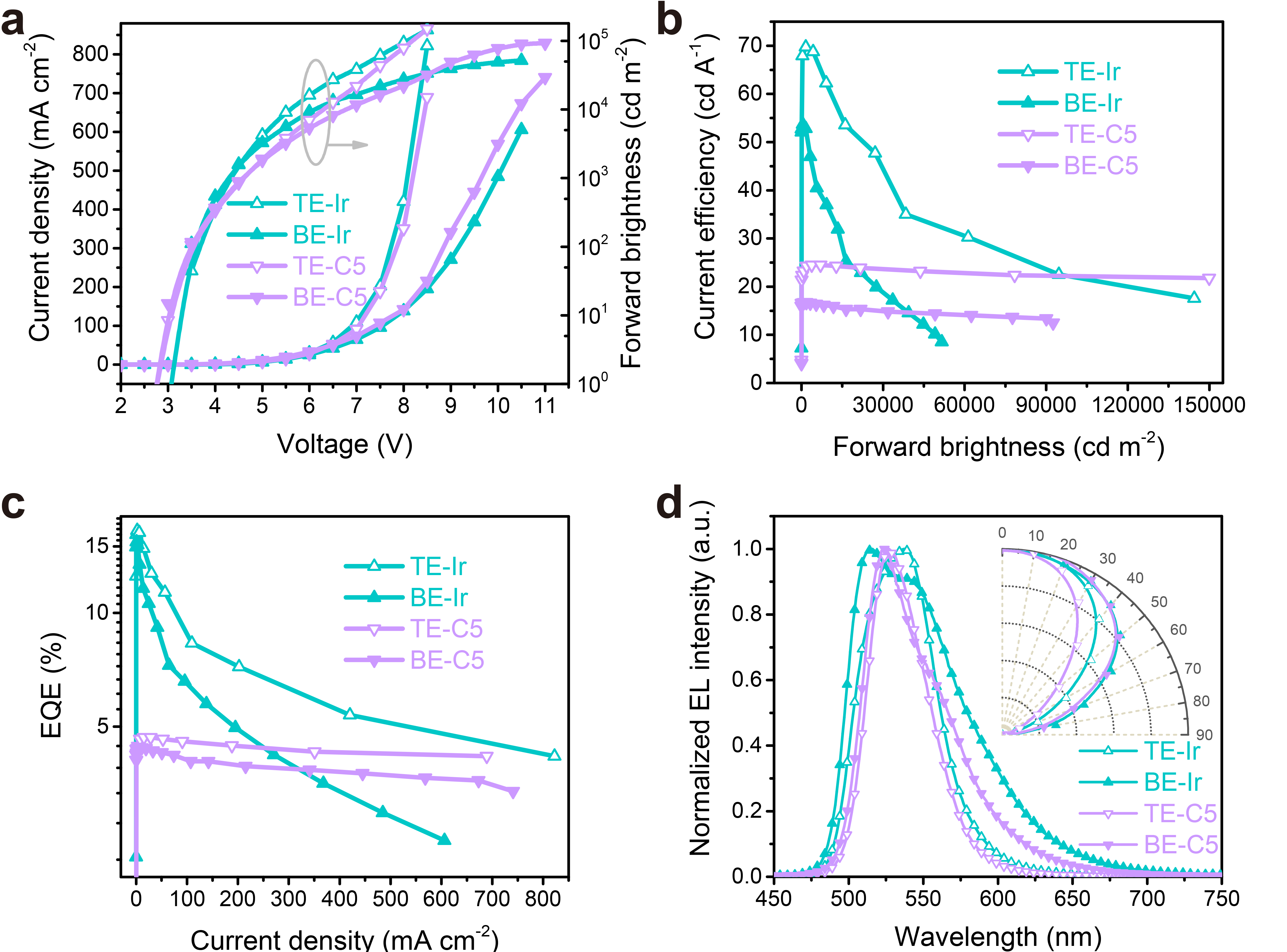


**Figure S6**. **(a)** Current density-voltage-forward brightness, **(b)** current efficiency-brightness, **(c)** EQE-current density and **(d)** normalized EL spectra characteristics of devices TE-C5, TE-Ir, BE-C5 and BE-Ir.


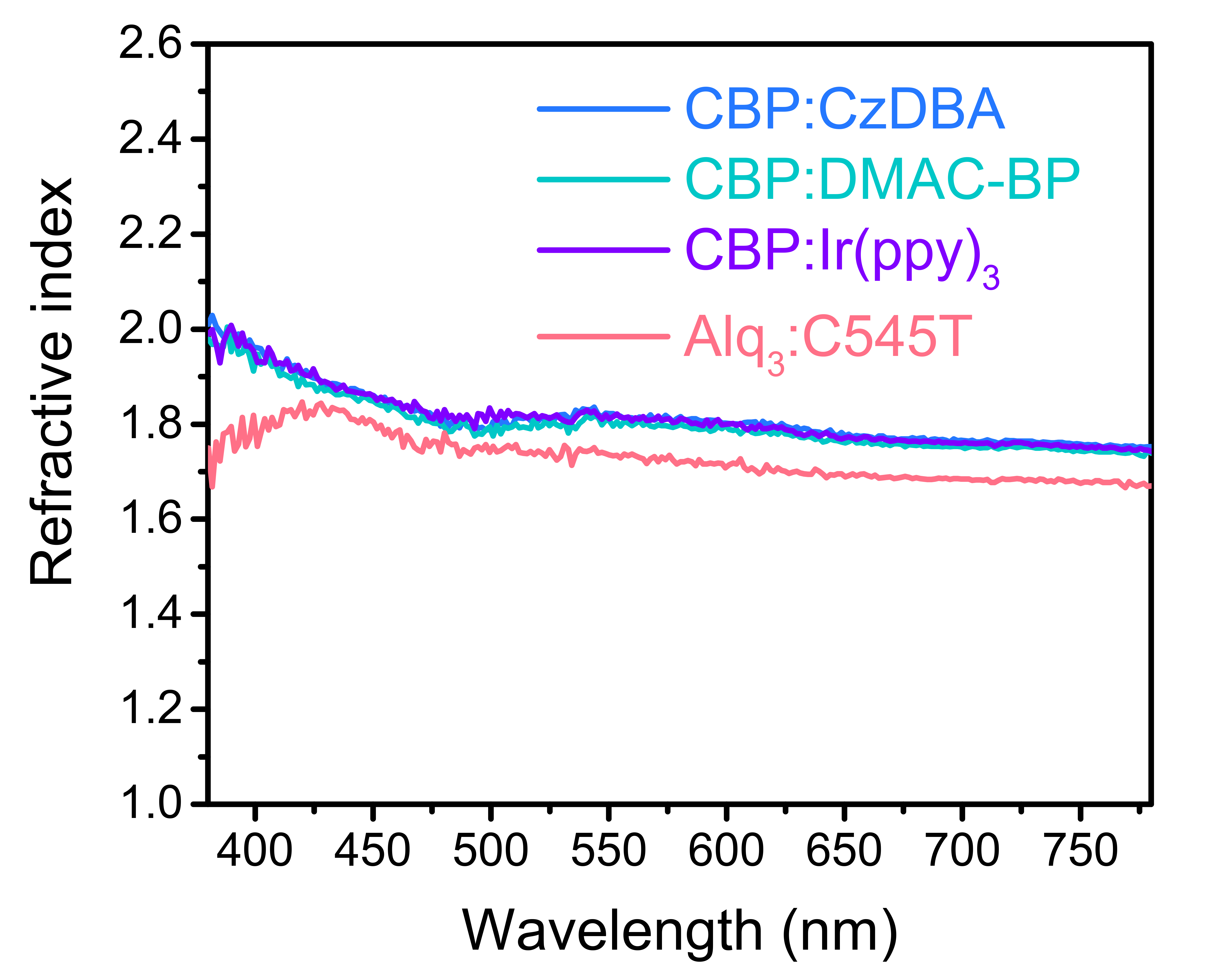


**Figure S7**. Refractive indexes of the emitting layers used in the top-emitting and conventional devices. The emitting layers are prepared by doping 10 wt% guest emitters into their host materials.

**Supplementary Note 2:**

Purcell factor F(λ) is also known as the total radiated power at the emitter location in the framework of the dipole model. The spectral power F(λ) quantifies the effect of the optical environment on the emissive properties of molecular species and therefore is the classical equivalent of the quantum-mechanical photonic mode density ^[S3]^. The total radiated power F(λ) is equal to the integral of the radiating power densities K(λ, u) of dipole sources ^[S3]^:

$F\left( \lambda\right)=\int_{0}^{\infty} K\left( \lambda, u \right)du^{2}=\int_{0}^{\infty} [(1-\Theta)\cdot K_{TMv}+\Theta\cdot(K_{TMh}+K_{TEh})]du^{2}$.

Where, Θ is the horizontal dipole ratio, and u is unit normalized transverse wave vector. The three components of the total power density, namely K_TMv_ for vertical dipoles coupling to TM waves, and K_TMh_ and K_TEh_ for horizontal dipoles coupling to TM and TE waves, respectively, can be calculated separately:

$K_{TMv}=\frac{3}{4}Re[\frac{u^{2}}{\sqrt{1-u^{2}}}\frac{(1+a_{TM}^{+})(1+a_{TM}^{-})}{1-a_{TM}}]$,

$K_{TMh}=\frac{3}{8}Re[\sqrt{1-u^{2}}\frac{(1-a_{TM}^{+})(1-a_{TM}^{-})}{1-a_{TM}}]$,

$K_{TMv}=\frac{3}{4}Re[\frac{1}{\sqrt{1-u^{2}}}\frac{(1+a_{TE}^{+})(1+a_{TE}^{-})}{1-a_{TE}}]$.

Where, Re[] denotes the real part of the complex. Moreover, $a_{TM, TE}^{+}=r_{TM, TE}^{+}exp(2jk_{z,e}z^{+})$,$a_{TM, TE}^{-}=r_{TM, TE}^{-}exp(2jk_{z,e}z^{-})$, and $a_{TM, TE}=a_{TM, TE}^{+}a_{TM, TE}^{-}$. Here, $r_{TM, TE}^{+}$ ($r_{TM, TE}^{-}$) represents the reflection coefficient for waves traveling from the emitting layer in the upward (downward) direction for TM and TE polarized waves; z^+^ (z^−^) is the distance of the emitting dipoles from the interface between the top (bottom) electrode and the active layers; k_z,e_ is the out-of-plane component of the wavevector for propagation in the emitting layer.


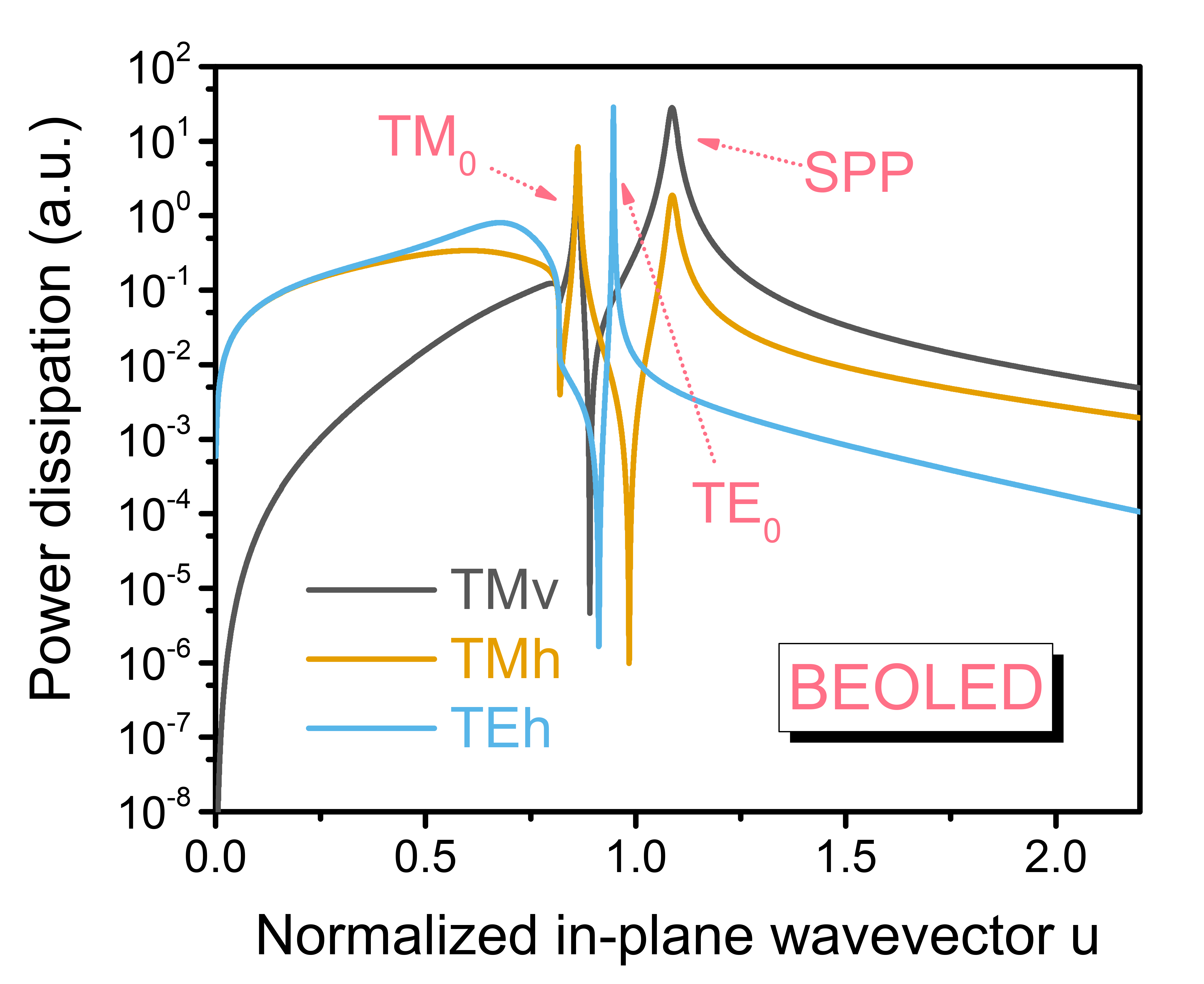


**Figure S8**. Power dissipation spectra at λ=540 nm for the conventional bottom-emitting devices. The in-plane wavevector is normalized with respect to propagation in the emitting layer. The spectra are shown for the vertical dipole (TMv) and for the horizontal dipole coupling to TM and TE cavity modes (TMh and TEh, respectively).


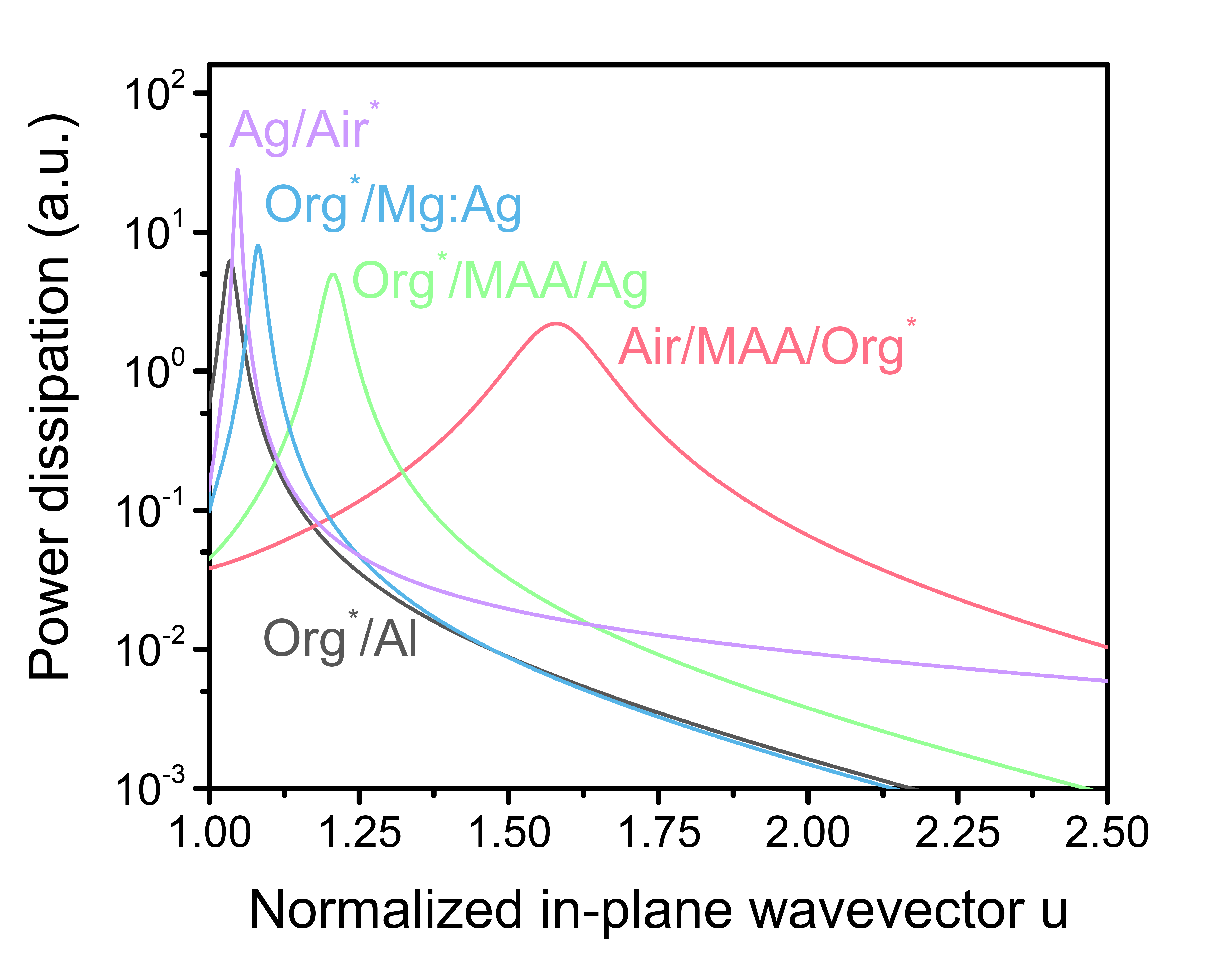


**Figure S9**. Power dissipation spectra at λ=540 nm for the multilayer structures: organic (Org)/Al (120 nm), Org/Mg:Ag (120 nm), Org/MAA/Ag (100 nm), Ag (120 nm)/Air and Air/MAA/Org, and the emitting layer is located in the medium marked with *.


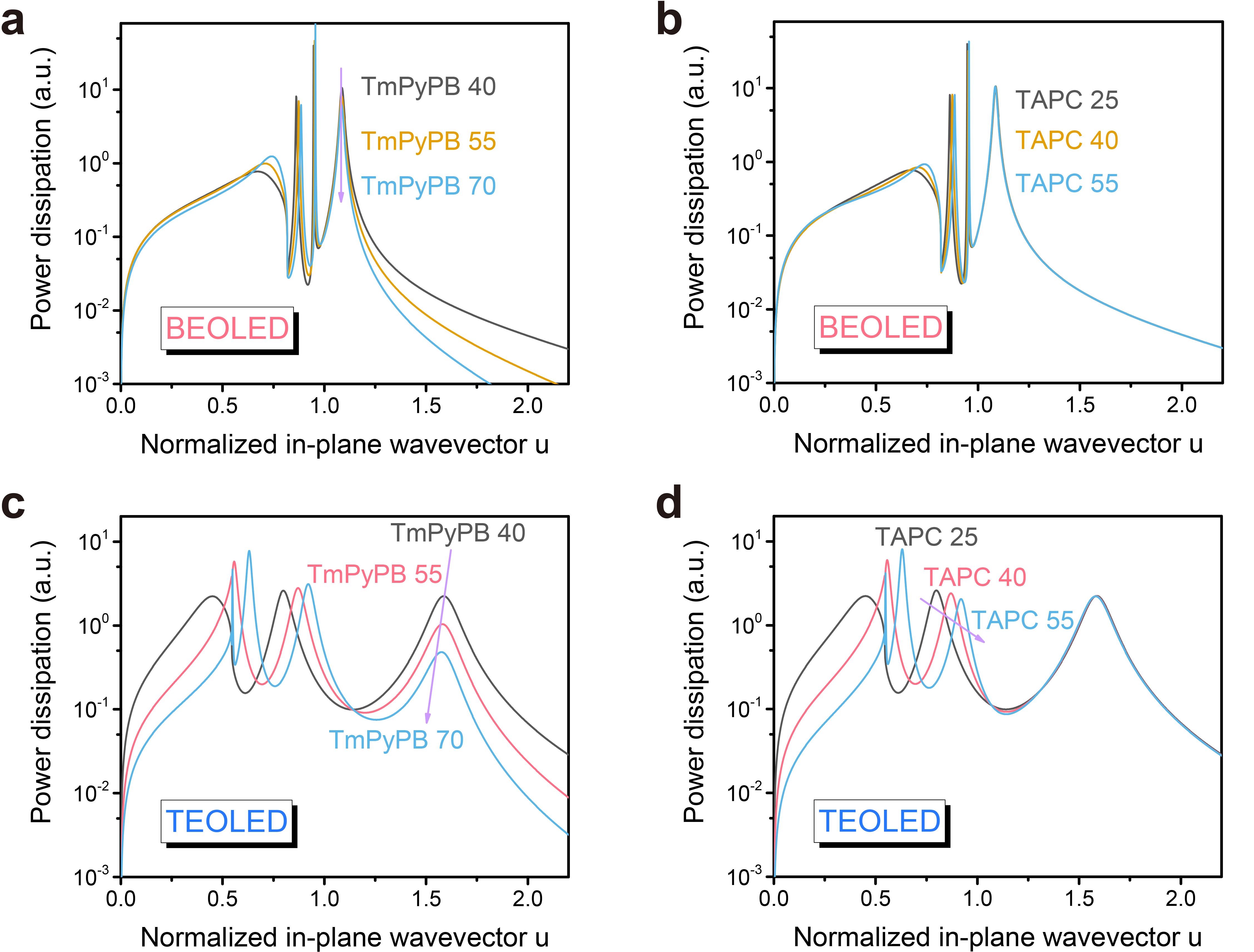


**Figure S10**. Power dissipation spectra at λ=540 nm for different OLED structures. **(a)** is for the conventional bottom-emitting devices with variable TmPyPB (40, 55 and 70 nm). **(b)** is for the conventional devices with variable TAPC (25, 40 and 55 nm). **(c)** is for the top-emitting devices with variable TmPyPB (40, 55 and 70 nm). **(d)** is for the top-emitting devices with variable TAPC (25, 40 and 55 nm).

**Supplementary Note 3:**

Surface plasmon polaritons (SPPs) are electromagnetic waves that travel along a metal/dielectric or metal/air interface. An SPP is a purely evanescent wave in the out-of-plane direction so that its wave vector contains an out-of-plane component with a pure imaginary value. It means that an SPP has a high wavevector (k) in the in-plane direction, and thus always has high momentum (ћk) than a free-space photon with a same frequency. Coupling of photons into SPPs can only be achieved when they have the same frequency and momentum ^[S4-S5]^. Thus, a free-space photon cannot couple directly to an SPP. Nevertheless, the dipole radiation in OLEDs belongs to a near-field radiation in a region within a radius r ≪ λ. Due to the uncertain principle, the radiation has an uncertain momentum in the presence of a limited length scale. Power components of the dipole radiation can be characterized by values of the in-plane component of the normalized wavevector larger than unity (u>1). In this region, coupling of photons into SPPs can be achieved, and the radiated power components are transferred to SPPs. As a result, a peak corresponding to the SPP at the organic/Mg:Ag interface is observed at u~1.08 in the power dissipation spectra (red line in Figure 3a and blue line in Figure S9). Because the SPP peak is caused by the coupling of the near-field radiation into SPPs, the intensity of the SPP peak will decrease as the distance between the dipole radiation and the metal/organic interface increases, as shown in Figure S10a and S10b.

In the top-emitting devices, there are three metal/dielectric or metal/air interfaces, including the air/MAA, MAA/organic and organic/Al interfaces. However, only two peaks are observed in the power dissipation spectra (blue line in Figure 3a) of the top-emitting devices. As shown in Figure S9, the two peaks (blue line in Figure 3a) cannot correspond to the coupling of the dipole radiation into a single metal/organic or metal/air interface. But the peak at u~1.59 corresponds to the hybrid coupling of the dipole radiation into the organic/MAA and MAA/air interfaces (red line in Figure S9). Besides, as the thickness of the electron transport layer (the distance between the dipole radiation and the MAA cathode) increases, the intensity of the peak at u~1.59 decreases (Figure S10c). As the thickness of the hole transport layer (the distance between the dipole radiation and the Al anode) increases, the intensity of the peak at u~0.8 decreases and its peak position shifts as well (Figure S10d). It’s thus reasonably considered that the peak at u~0.8 corresponds to a hybrid mode of the SPP at organic/Al interface and the Fabry-Pérot (FP) cavity. As a result of the confinement of an SPP in an optical cavity of size smaller than the wavelength of light used to excite the plasmon, coupling of the dipole ration to SPP is possible in the region u<1 ^[S4, S5]^.


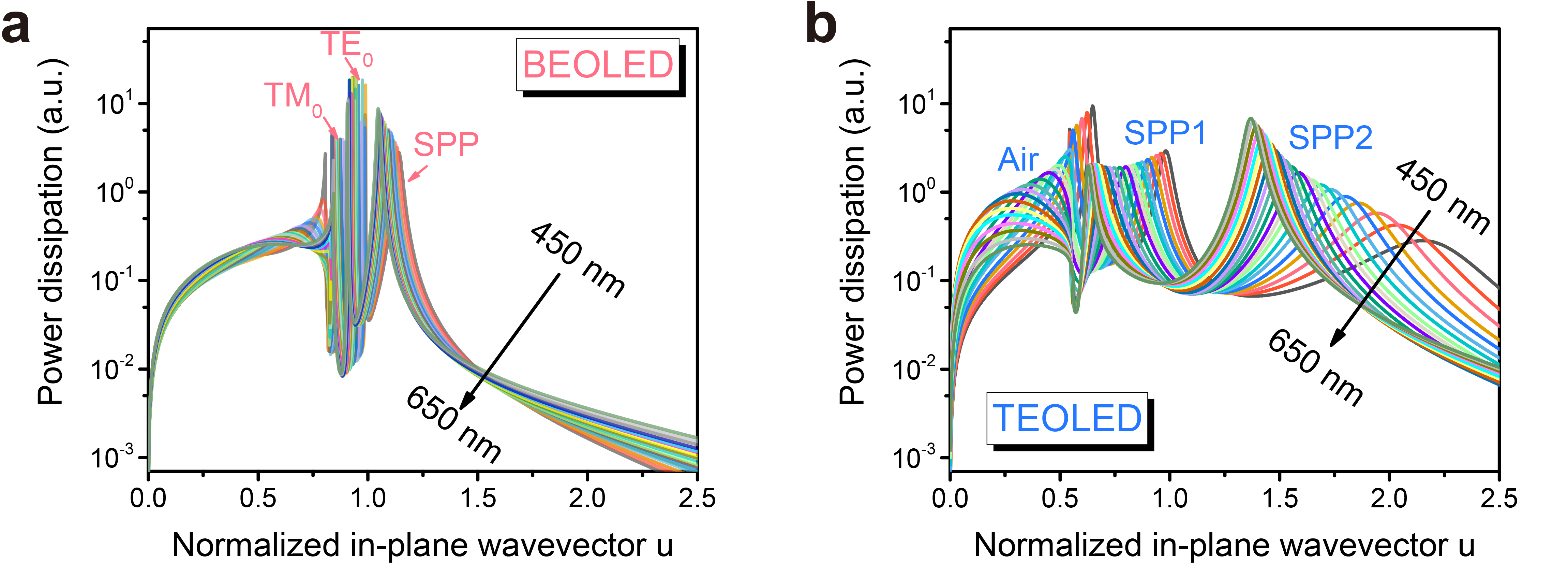


**Figure S11**. Power dissipation spectra at different wavelengths for the conventional devices and the top-emitting devices.


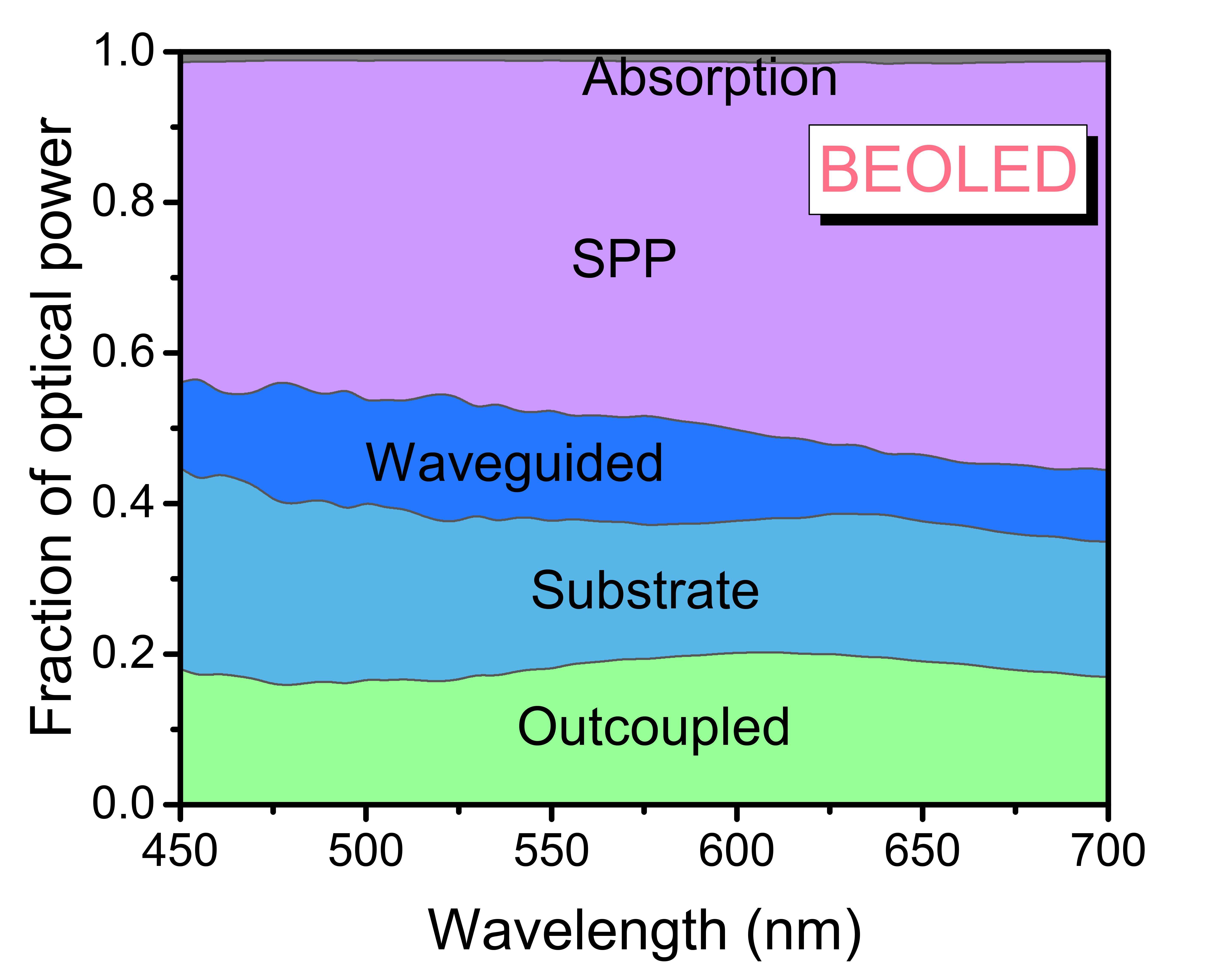


**Figure S12**. Distribution of all optical loss channels in the conventional devices.


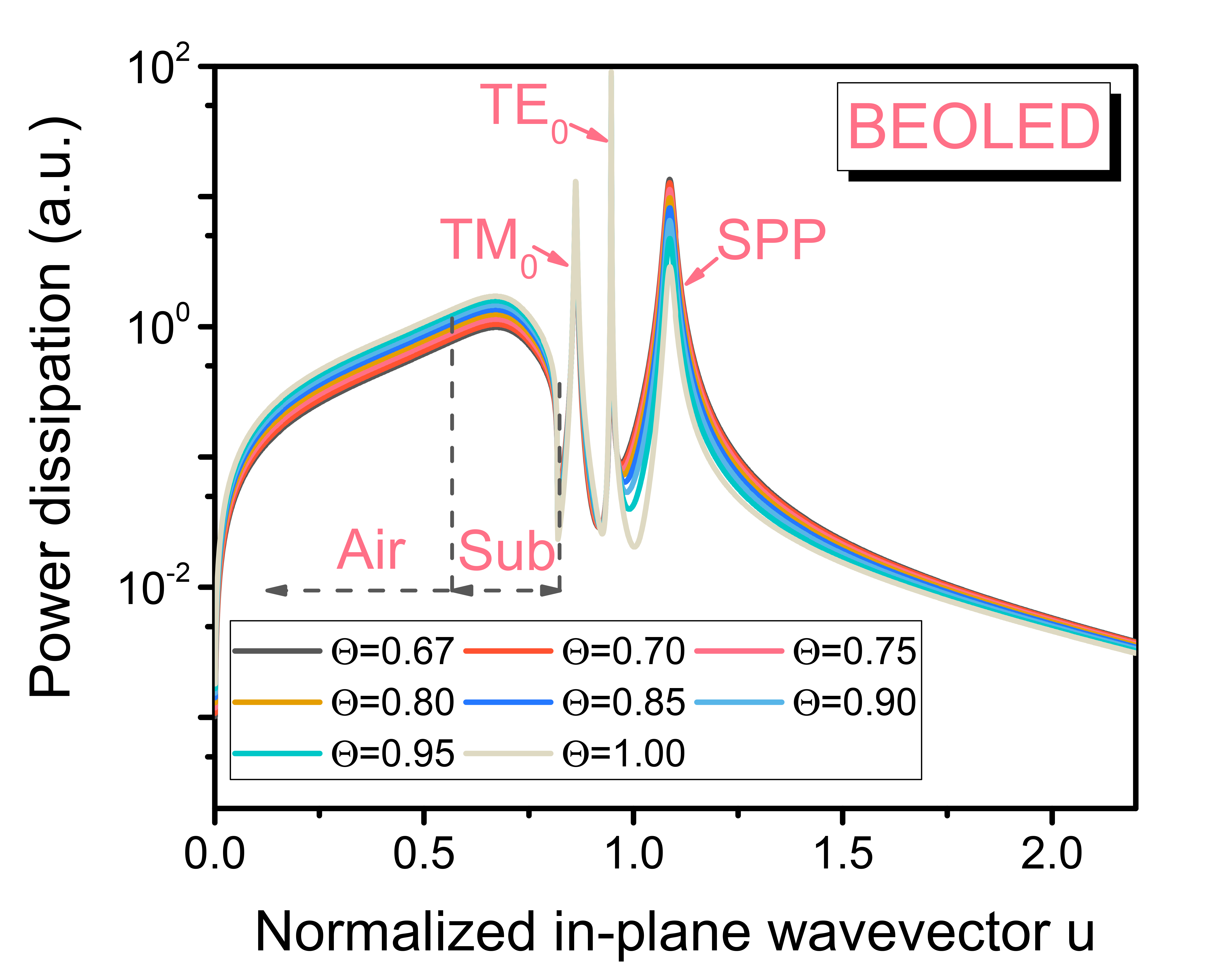


**Figure S13**. Power dissipation spectra of organic emitters with different horizontal dipole ratios Θ in the conventional devices.


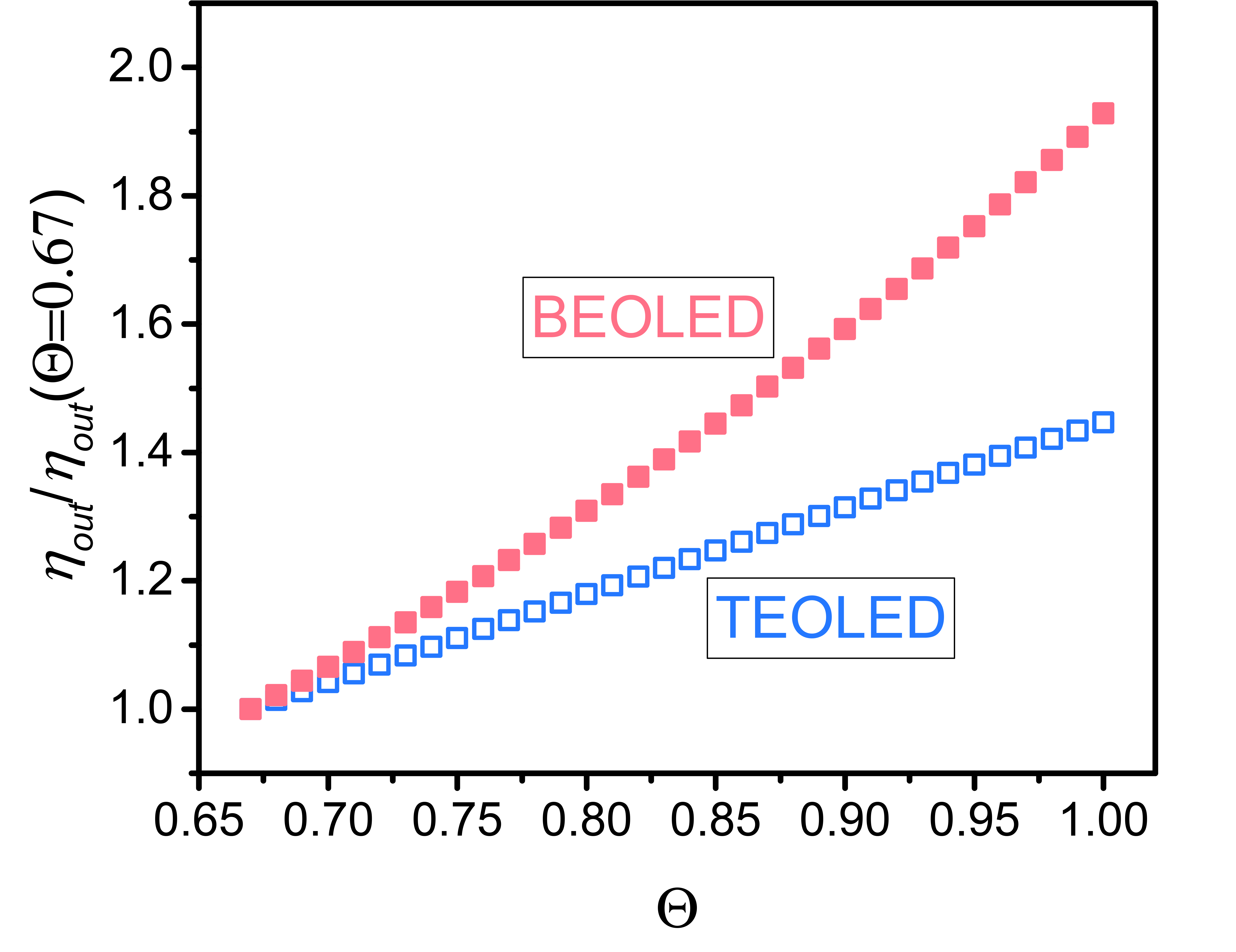


**Figure S14**. Ratio of the outcoupling efficiency η_out_ of organic emitter with different horizontal dipole ratios Θ at 540 nm to the outcoupling efficiency η_out_ (Θ=0.67) of organic emitter with Θ=0.67 at 540 nm in the conventional devices and the top-emitting devices.


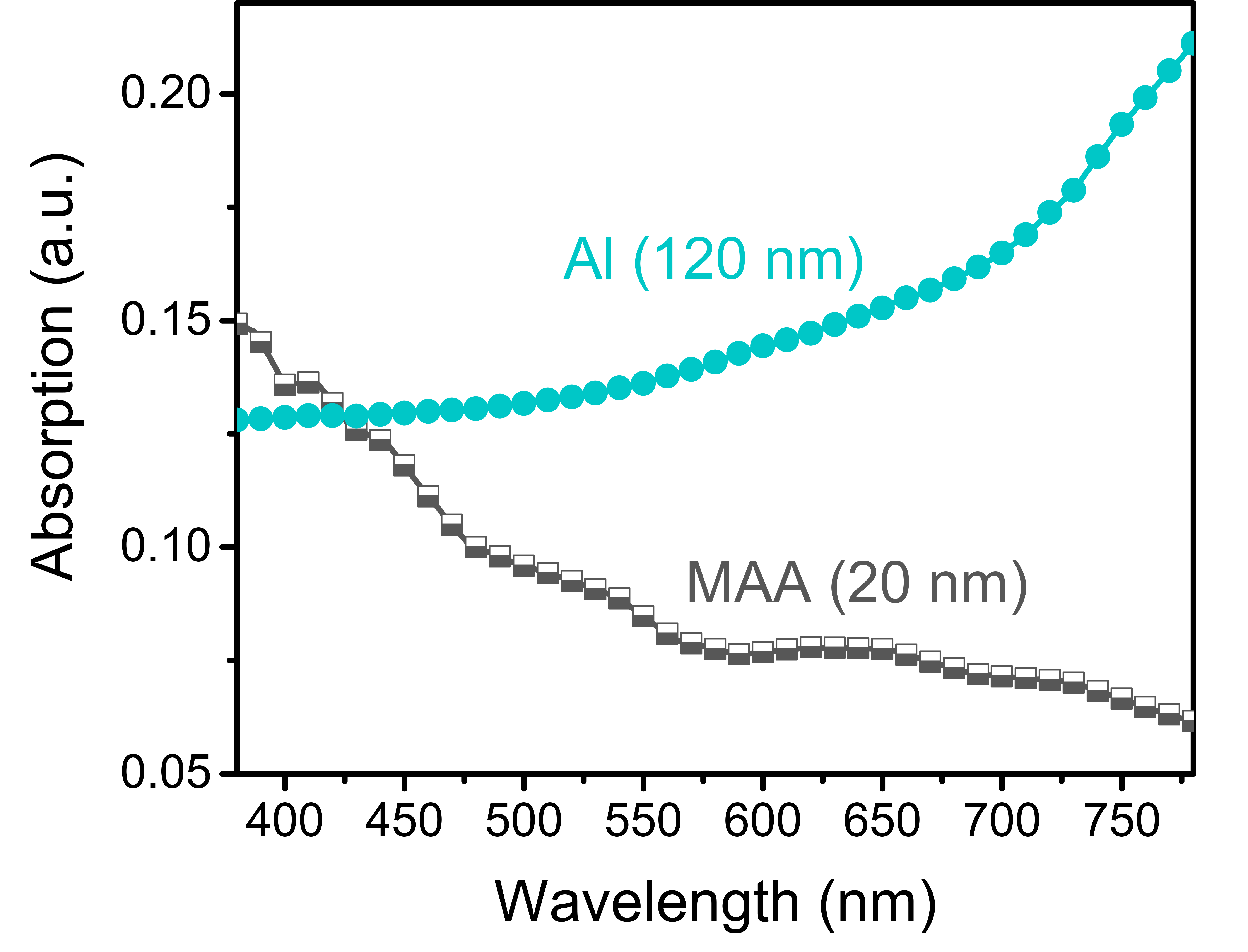


**Figure S15**. Absorption characteristics of the MAA (20 nm) and Al (120 nm) layers.


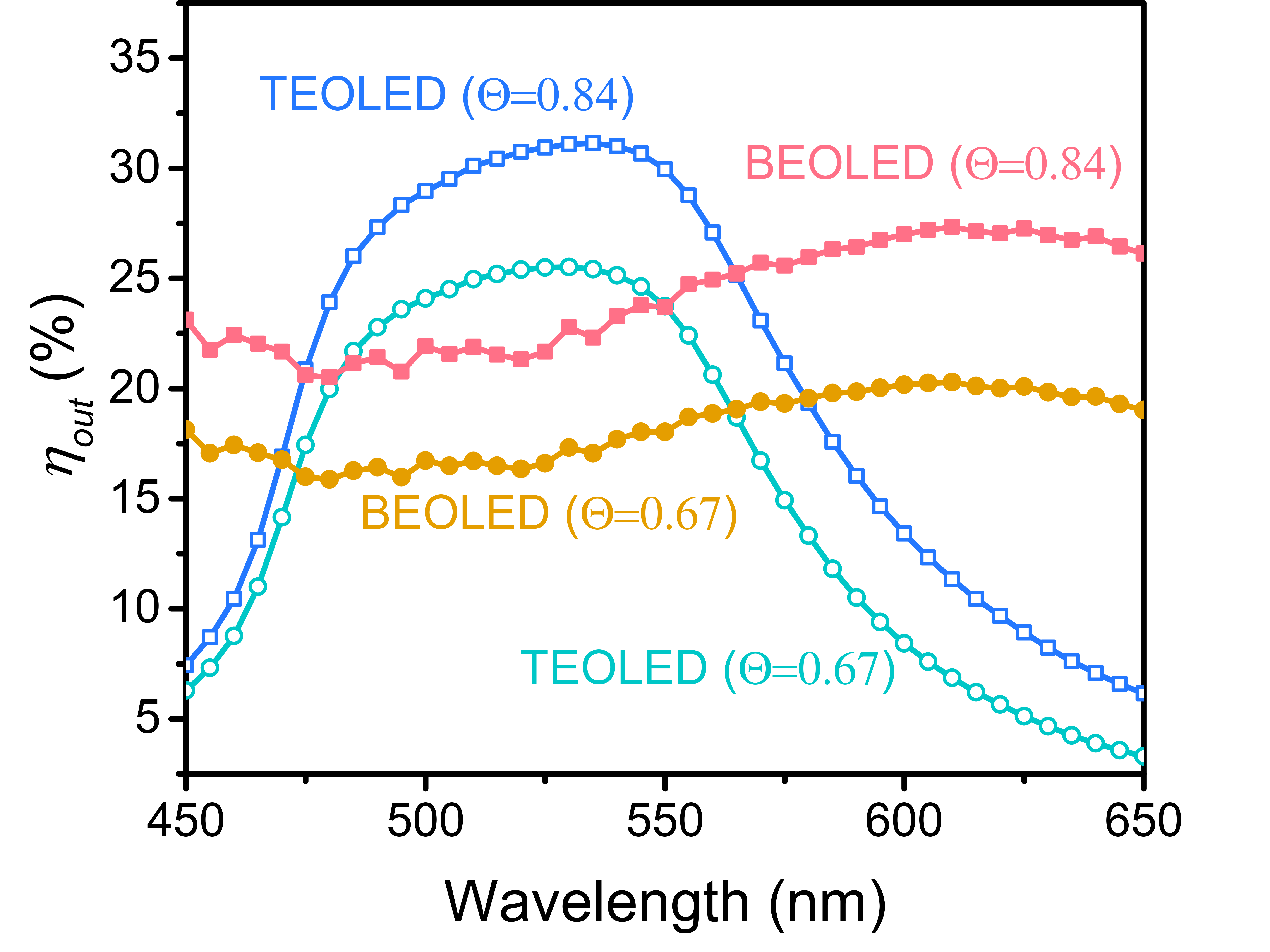


**Figure S16**. Outcoupling efficiency of organic emitters with Θ=0.84 and Θ=0.67 at different wavelengths in the conventional devices and the top-emitting devices.


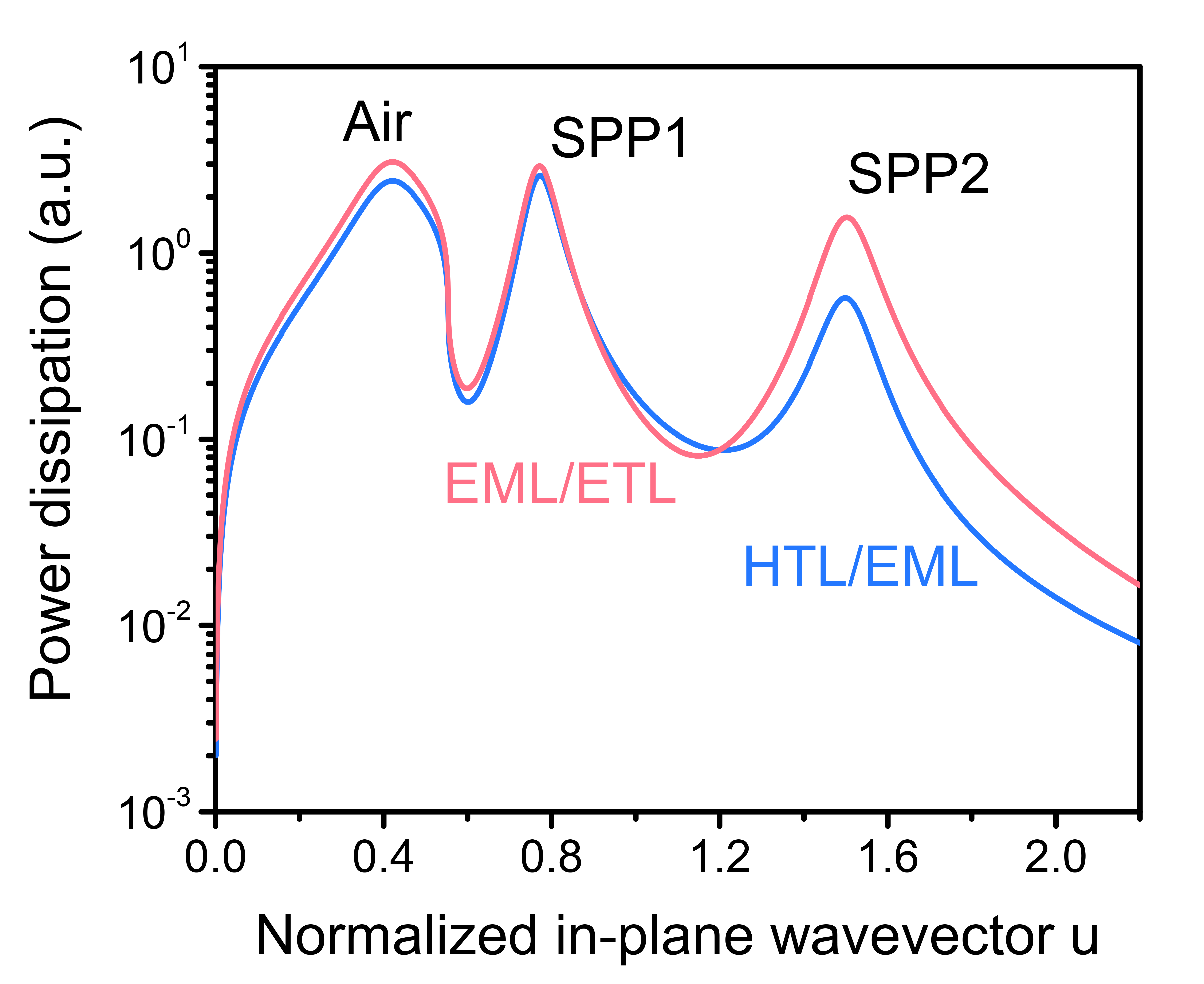


**Figure S17.** Power dissipation spectra for dipole sources at the EML/ETL interface and the HTL/EML interface.


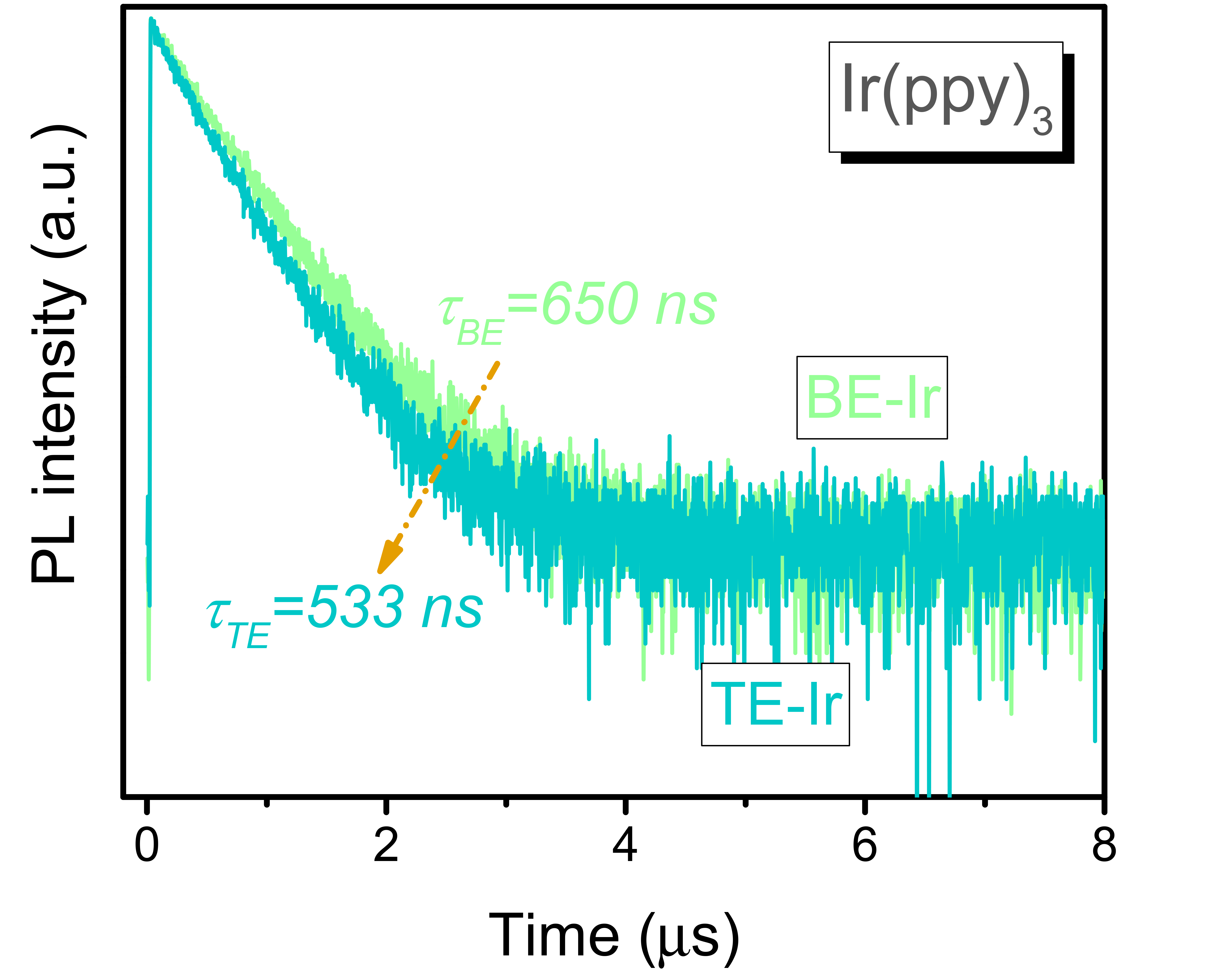


**Figure S18**. Transient PL decay characteristics of Ir(ppy)_3_ in the devices TE-Ir and BE-Ir.


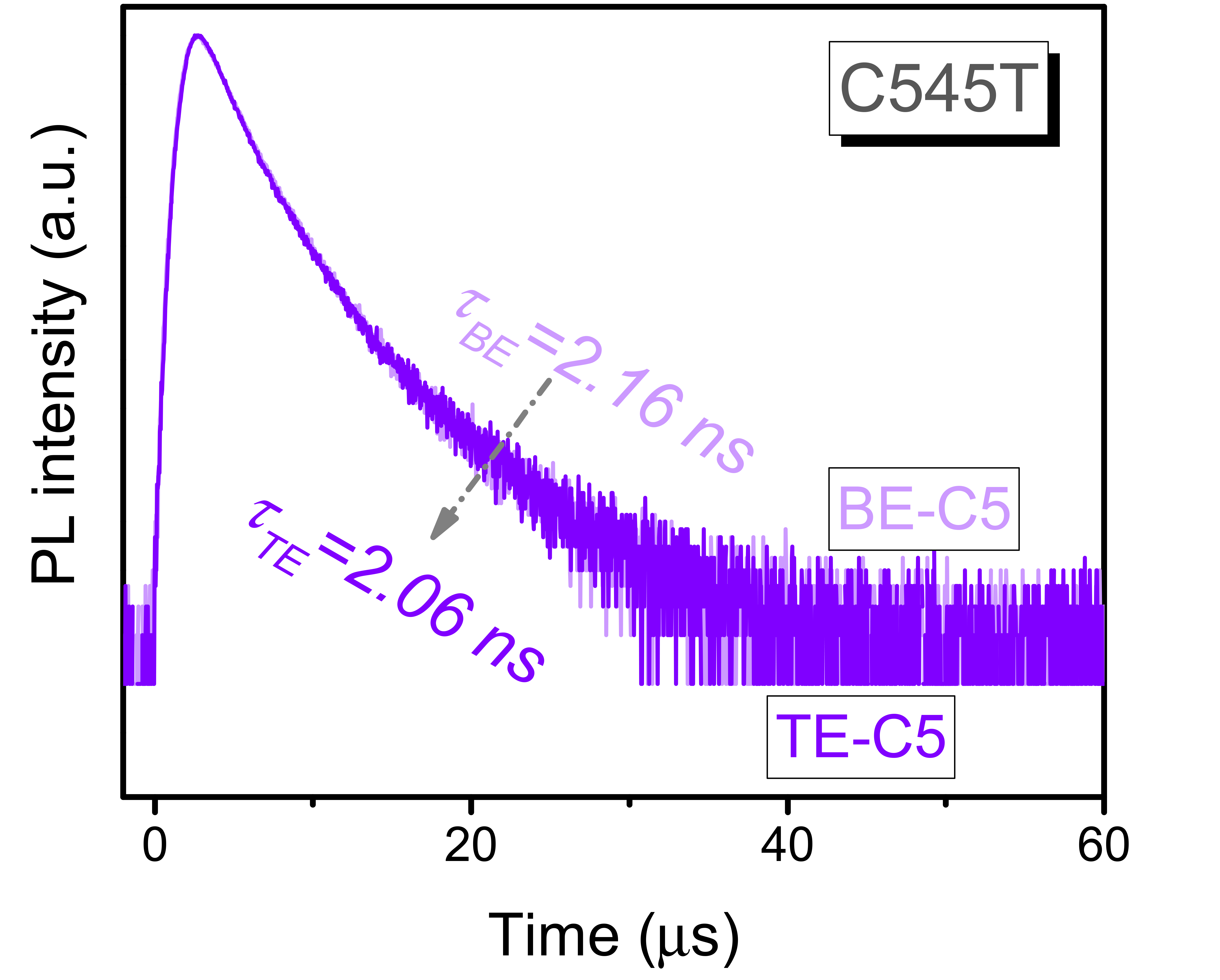


**Figure S19**. Transient PL decay characteristics of C545T in the top-emitting devices and the conventional devices.


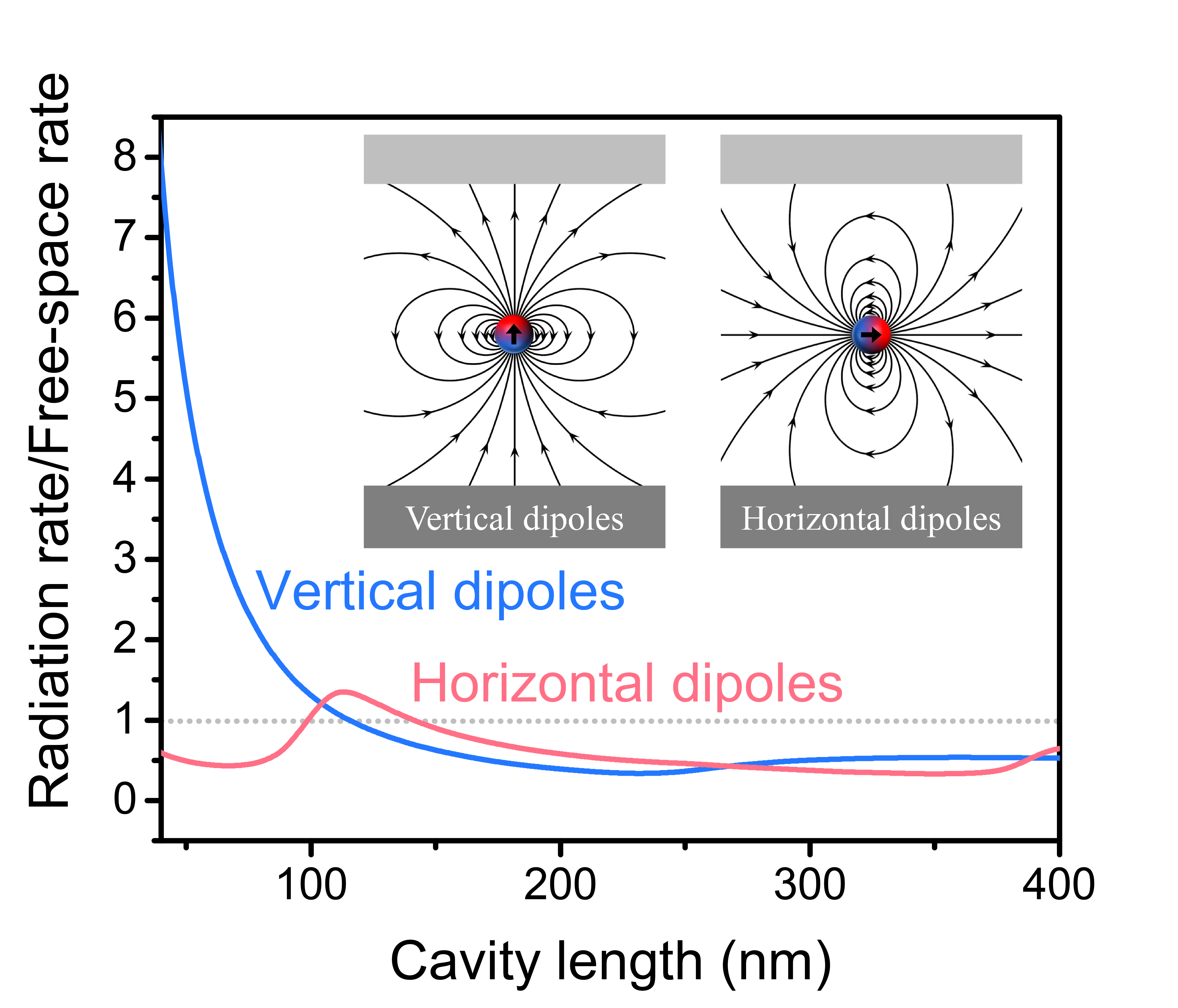


**Figure S20.** Radiation rate of dipole sources in devices with different cavity lengths. The insets are radiation patterns of electrical dipoles.


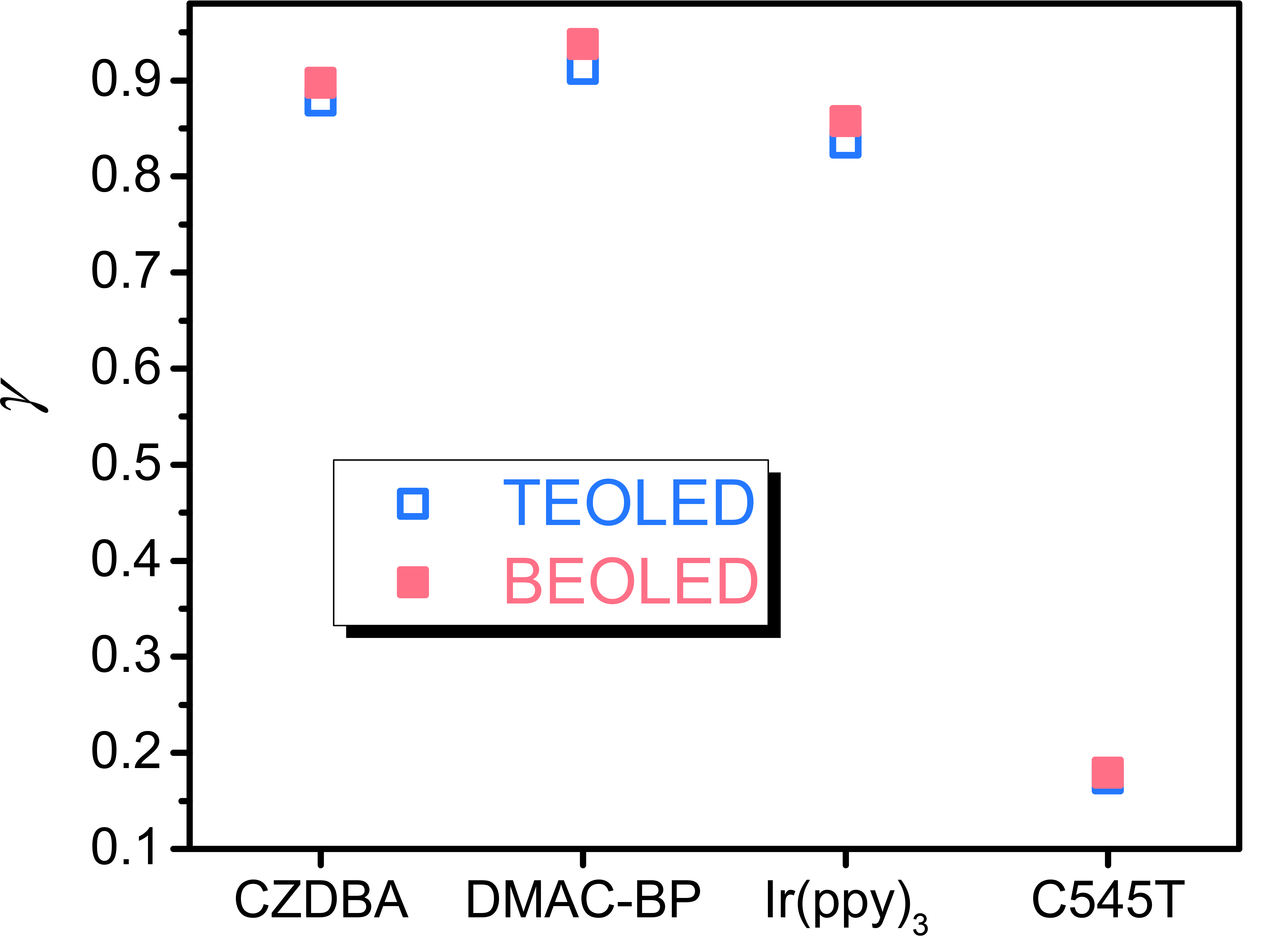


**Figure S21**. Ratio, γ, of generated excitons (singlets for C545T) to the number of injected electron-hole pairs.


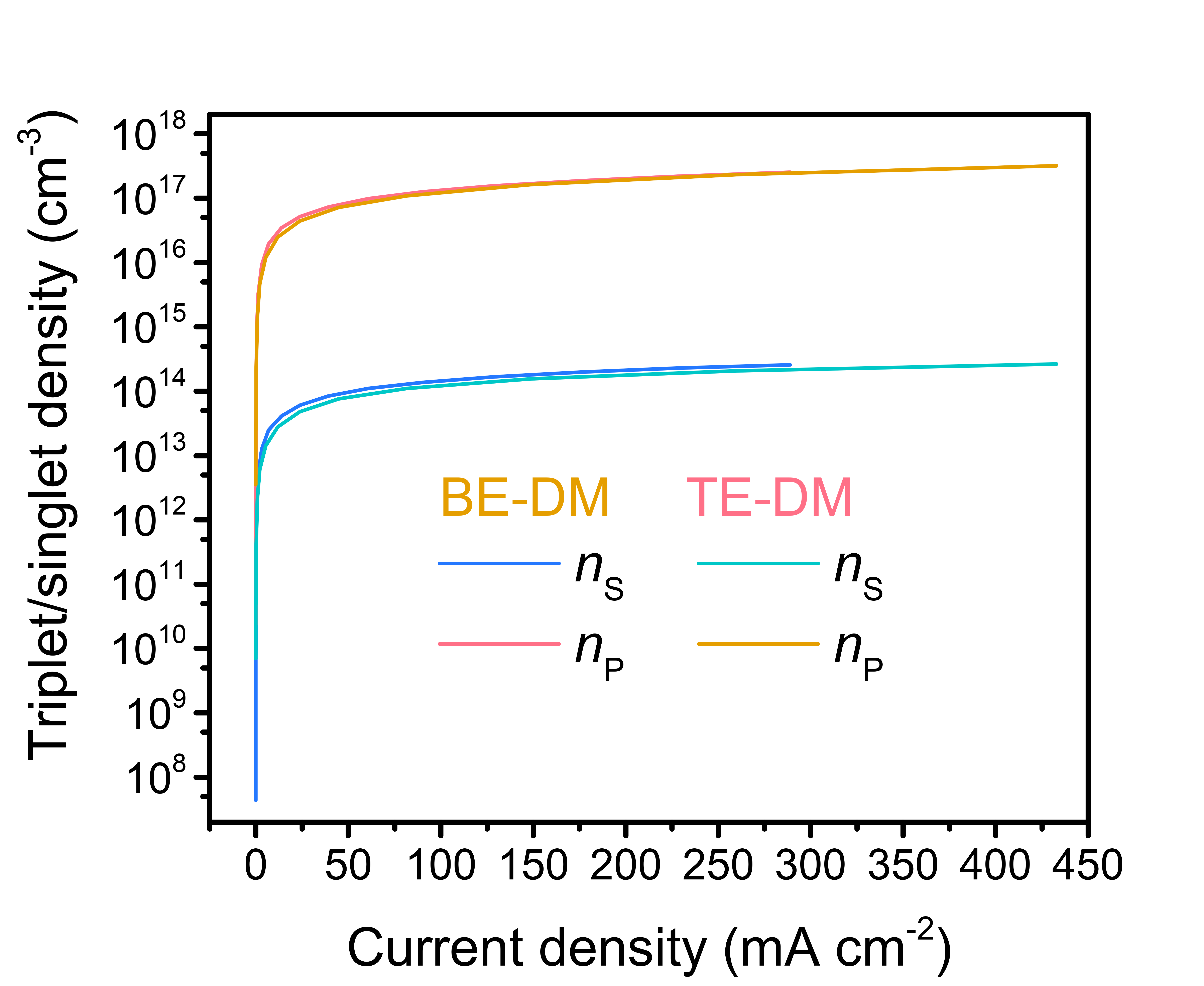


**Figure S22**. Triplet/singlets densities of devices BE-DM and TE-DM at different current densities.


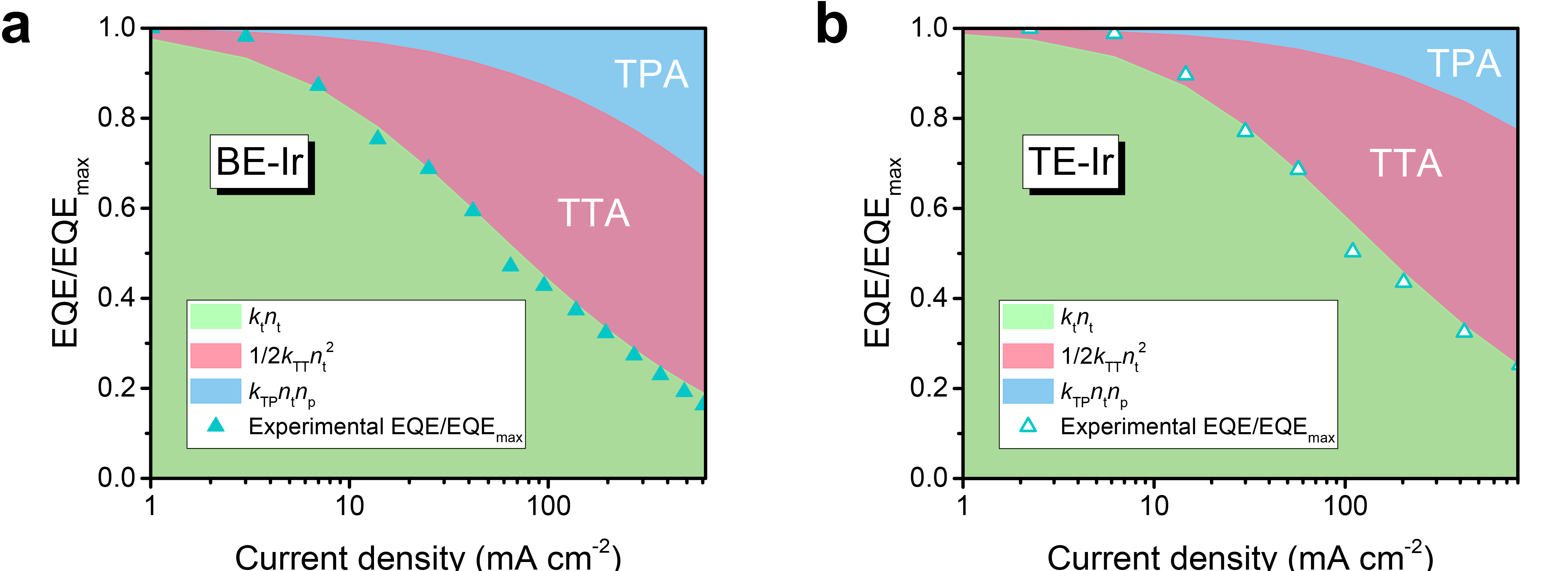


**Figure S23**. Theoretically calculated contributions of triplet-triplet annihilation (TTA) and polaron-triplet annihilation (TPA) to EQE loss in **(a)** device BE-Ir and **(b)** device TE-Ir as a function of current density. Experimentally measured relative EQE is shown as the symbol of empty and solid triangles.


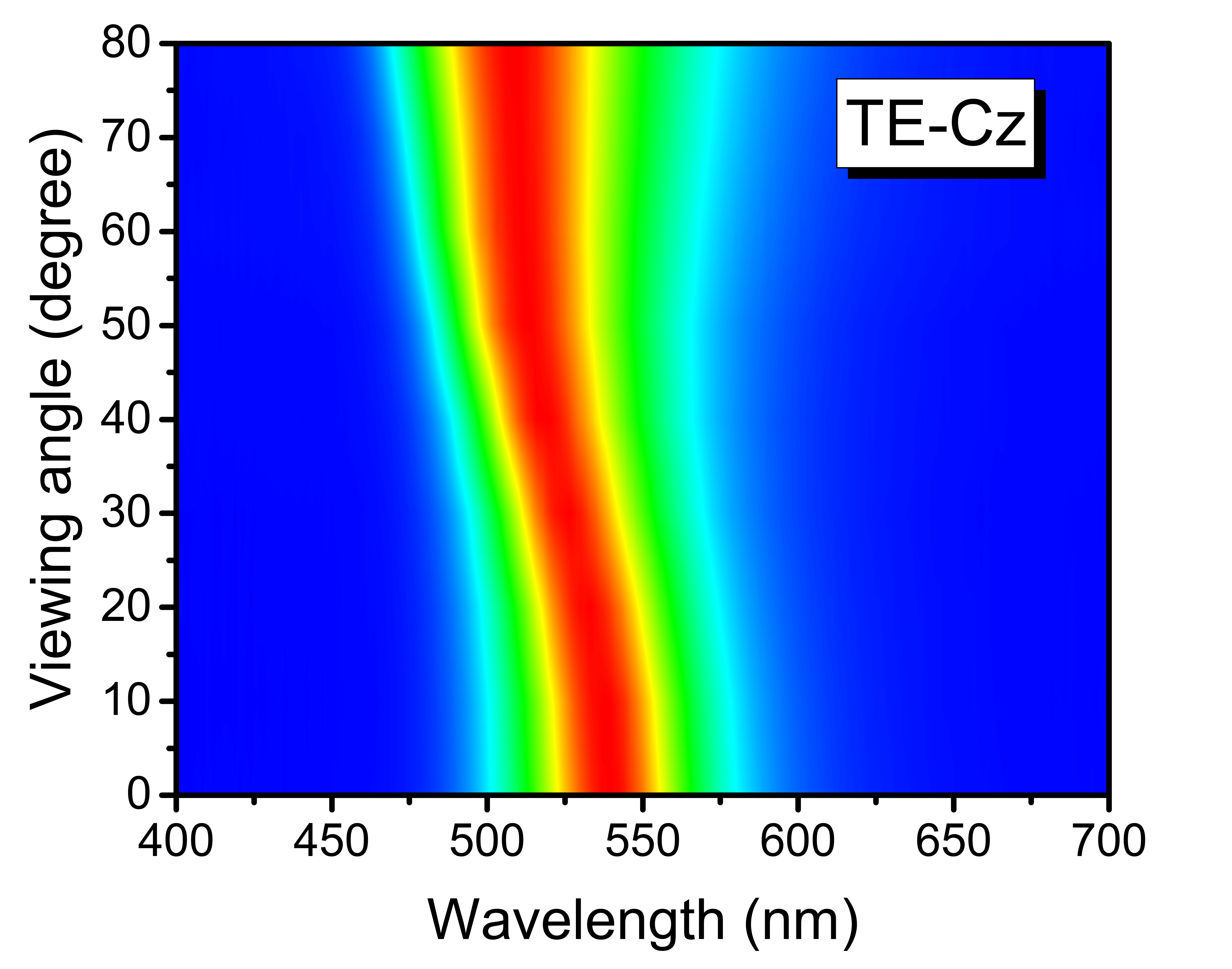


**Figure S24**. Spectral power distribution of device TE-Cz at different viewing angles.


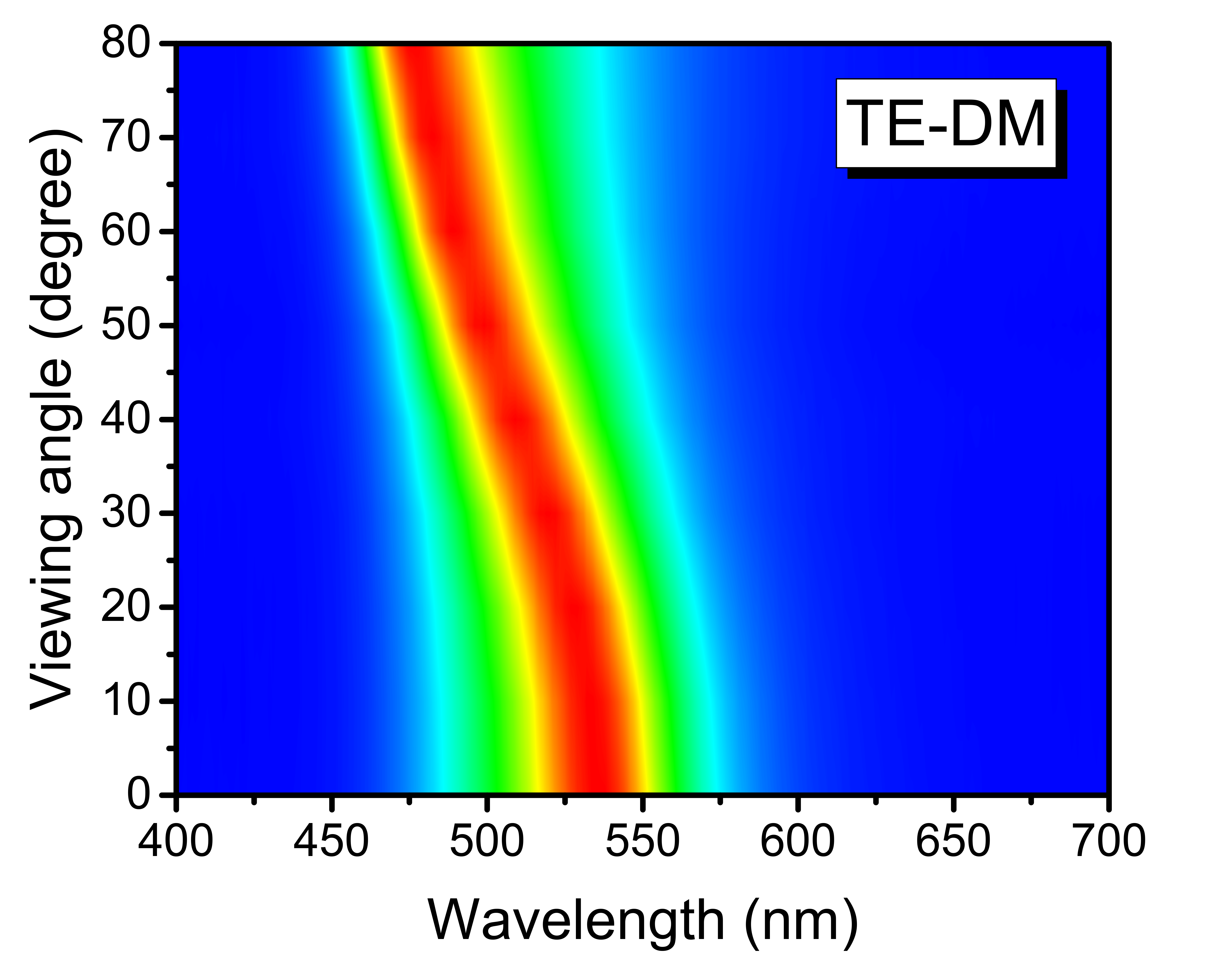


**Figure S25**. Spectral power distribution of device TE-DM at different viewing angles.

**Table S1.** Device performances of the top-emitting devices and the bottom-emitting devices

| Emitter | Device type | Max. CE (cd A^-1^) | CE@5000 cd m^-2^  (cd A^-1^) | Max. EQE  (%) | FHWM  (nm) | Color purity (%) |
| --- | --- | --- | --- | --- | --- | --- |
| CzDBA | TE | 108.2 | 92.3 | 21.3 | 51 | 88.0 |
|  | BE | 73.5 | 58.2 | 21.5 | 93 | 81.8 |
| DMAC-BPP | TE | 68.1 | 52.6 | 16.5 | 55 | 71.4 |
|  | BE | 42.5 | 21.8 | 14.0 | 98 | 43.4 |
| C545T | TE | 24.5 | 24.5 | 4.7 | 46 | 90.1 |
|  | BE | 16.7 | 16.4 | 4.4 | 60 | 89.4 |
| Ir(ppy)_3_ | TE | 69.7 | 67.7 | 16.5 | 60 | 90.0 |
|  | BE | 53.7 | 42.2 | 15.4 | 82 | 66.7 |

**Table S2.** Radiative efficiencies η_rad_ and η_rad’_ with and without the Purcell effect

|  | CzDBA | DMAC-BP | Ir(ppy)_3_ | C545T |
| --- | --- | --- | --- | --- |
| η_rad_ | 100%^[S6]^ | 86%^[S7]^ | 100%^[S8]^ | 95%^[S9]^ |
| η_rad’_ | 100% | 91% | 100% | 95% |

**Table S3**. Parameters used in the calculation for the EQE loss.

|  | BE-DM | TE-DM | BE-Ir | TE-Ir |
| --- | --- | --- | --- | --- |
| k_L_ (cm^-3^ s^-1^) | 4.5×10^-10^ | 4.5×10^-10^ | 4.5×10^-10^ | 4.5×10^-10^ |
| k_S_ or ks’ (s^-1^) | 3.6×10^7^ | 5.6×10^7^ | n/a | n/a |
| k_ST_ (cm^-3^ s^-1^) | 3×10^-10^ | 3×10^-10^ | n/a | n/a |
| k_TT_ (cm^-3^ s^-1^) | 9×10^-13^ | 9×10^-13^ | 5×10^-11^ | 5×10^-11^ |
| k_T_ (s^-1^) | 1.2×10^4^ | 1.2×10^4^ | 1.5×10^6^ | 1.9×10^6^ |
| k_TP_ (cm^-3^ s^-1^) | n/a | n/a | 3.8×10^-12^ | 3.8×10^-12^ |
| k_ISC_ (s^-1^) | 1.6×10^7^ | 2.5×10^7^ | n/a | n/a |
| k_RISC_ (s^-1^) | 4.6×10^5^ | 4.6×10^5^ | n/a | n/a |

**Reference:**

S1. Deppe, D. et al. Spontaneous emission from planar microstructures. Journal of Modern Optics **41**, 325-344 (1994).

S2. Hofmann, S. et al. Top-emitting organic light-emitting diodes. Optics Express **19**, A1250-A1264 (2011).

S3. Furno, M. et al. Efficiency and rate of spontaneous emission in organic electroluminescent devices. Physical Review B **85**, 115205 (2012).

S4. Yamamoto, Y. et al. Semiconductor cavity quantum electrodynamics, Springer: 2003; Vol. 169.

S5. Haroche, S. et al. Cavity quantum electrodynamics. Physics Today **42**, 24-30 (1989).

S6. Wu, T.-L. et al. Diboron compound-based organic light-emitting diodes with high efficiency and reduced efficiency roll-off. Nature Photonics **12**, 235-240 (2018).

S7. Wong, M. Y. et al. Purely organic thermally activated delayed fluorescence materials for organic light-emitting diodes. Advanced Materials **29**, 1605444 (2017).

S8. Kim, K. H. et al. Highly efficient organic light-emitting diodes with phosphorescent emitters having high quantum yield and horizontal orientation of transition dipole moments. Advanced Materials **26**, 3844-3847 (2014).

S9. Benor, A. et al. Efficiency improvement of fluorescent OLEDs by tuning the working function of PEDOT: PSS using UV–ozone exposure. Organic Electronics **11**, 938-945 (2010).
